# Is there a maternal blood biomarker that can predict spontaneous preterm birth prior to labour onset? A systematic review

**DOI:** 10.1371/journal.pone.0265853

**Published:** 2022-04-04

**Authors:** Kylie K. Hornaday, Eilidh M. Wood, Donna M. Slater

**Affiliations:** 1 Department of Physiology and Pharmacology, Cumming School of Medicine, University of Calgary, Calgary, Alberta, Canada; 2 Department of Obstetrics and Gynecology, Cumming School of Medicine, University of Calgary, Calgary, Alberta, Canada; University of Cambridge, UNITED KINGDOM

## Abstract

**Introduction:**

The ability to predict spontaneous preterm birth (sPTB) prior to labour onset is a challenge, and it is currently unclear which biomarker(s), may be potentially predictive of sPTB, and whether their predictive power has any utility. A systematic review was conducted to identify maternal blood biomarkers of sPTB.

**Methods:**

This study was conducted according to PRISMA protocol for systematic reviews. Four databases (MEDLINE, EMBASE, CINAHL, Scopus) were searched up to September 2021 using search terms: “preterm labor”, “biomarker” and “blood OR serum OR plasma”. Studies assessing blood biomarkers prior to labour onset against the outcome sPTB were eligible for inclusion. Risk of bias was assessed based on the Newcastle Ottawa scale. Increased odds of sPTB associated with maternal blood biomarkers, as reported by odds ratios (OR), or predictive scores were synthesized. This review was not prospectively registered.

**Results:**

Seventy-seven primary research articles met the inclusion criteria, reporting 278 unique markers significantly associated with and/or predictive of sPTB in at least one study. The most frequently investigated biomarkers were those measured during maternal serum screen tests for aneuploidy, or inflammatory cytokines, though no single biomarker was clearly predictive of sPTB based on the synthesized evidence. Immune and signaling pathways were enriched within the set of biomarkers and both at the level of protein and gene expression.

**Conclusion:**

There is currently no known predictive biomarker for sPTB. Inflammatory and immune biomarkers show promise, but positive reporting bias limits the utility of results. The biomarkers identified may be more predictive in multi-marker models instead of as single predictors. Omics-style studies provide promising avenues for the identification of novel (and multiple) biomarkers. This will require larger studies with adequate power, with consideration of gestational age and the heterogeneity of sPTB to identify a set of biomarkers predictive of sPTB.

## Introduction

Globally, over 15 million babies are born prematurely each year, a value that, according to the Global Disease Burden study (2010–2019) has remained relatively consistent over the last decade. While neonatal mortality rates have dropped, largely attributed to advances in neonatal care, preterm birth continues to be associated with lifelong morbidities and is estimated to account for over 69 million disability-adjusted life years (DALYs) annually worldwide (Global Disease Burden Study, 2019). An estimated 45% of all preterm births occur following spontaneous onset of labour with no known maternal or fetal indications [1]. The etiology of a spontaneous preterm birth (sPTB) remains unclear, limiting strategies for prediction and management. Early prediction would allow for targeted clinical management, such as allowing time for the administration of progesterone or cervical cerclage and provide valuable reassurance to those identified as low risk for premature delivery.

Early prediction strategies are particularly limited for asymptomatic populations. Cervicovaginal levels of fetal fibronectin (fFN test) is used to predict imminent delivery (within seven days) [2]. The fFN test is commonly ordered for those 22–35 weeks pregnant with signs and symptoms of labour but is not generally recommended when asymptomatic for labour [3]. The strongest predictor of sPTB currently available is a previous preterm birth, which is associated with a 23% chance of another preterm birth [4]. While this can help guide clinical management in a subsequent pregnancy, this cannot help those at risk for a first-time sPTB. There is increasing evidence that biomarkers, or any biological entity or characteristic such as proteins or metabolites, in maternal circulation during pregnancy are associated with subsequent onset of sPTB [5, 6]. Biomarkers within amniotic fluid have been investigated for early prediction of sPTB [7], but amniocentesis can introduce risk of intrauterine infection and other unwanted complications [8]. There is an ongoing need for both more accessible and reliable tests for early prediction of sPTB, which would identify those at-risk and who may benefit from early intervention.

Peripheral blood is routinely collected during antenatal care and thus presents an alluring opportunity for a relatively non-invasive tool for prediction. This has garnered extensive studies on whether a blood test can accurately assess risk of subsequent preterm birth, including increasingly accessible high-throughput and -‘omics’ techniques, which have provided unprecedented amounts of data that can be used to predict health outcomes [9]. The aim of this review was to aggregate and synthesize existing data on biomarkers for spontaneous preterm birth, including to identify and synthesize biomarkers associated with or predictive of spontaneous preterm birth.

## Methods

### Research question

What biomarkers are predictive of, or associated with sPTB when there are no prior symptoms of labour?

### Review method

The search strategy, study selection, and data extraction protocols undertaken in this study were developed according to the PRISMA guidelines for systematic reviews [10]. The protocol for this study was not prospectively registered.

### Eligibility criteria

All observational studies, for example, primary case-control, cross-sectional or cohort studies were included. Literature reviews, systematic reviews with no meta-analysis, or incomplete publication (e.g. conference abstract) were excluded, as well as non-human studies. Eligible studies are those which investigated the association of maternal blood biomarkers for sPTB or developed a prediction model for sPTB using maternal blood biomarkers. Maternal samples included whole blood, plasma, or serum. Eligible biomarkers included biochemical and molecular biomarkers including, but not limited to, proteins, nucleic acids, or metabolites. Genetic and epi-genetic variant (single nucleotide polymorphisms or other) markers were excluded. At the time of sample collection, study participants must have no signs of labour, including but not limited to uterine contractions and rupture of membranes. Studies investigating risk of sPTB in multiple pregnancies, those that did not have a clear and standardized definition of sPTB as the outcome of interest, and provider initiated preterm births, otherwise known as medically indicated preterm births were also excluded.

### Search strategy

Four databases were searched for records: MEDLINE, EMBASE, CINAHL and Scopus. Additional studies were collected by searching reference lists of records and relevant review articles and using manual search of Google Scholar. The initial search was conducted on March 21, 2019, then repeated September 13th, 2021, to identify new publications. There was no time restriction to the search, though time frames vary by database (earliest date range 1788 Scopus– 1974 EMBASE). Only studies published in English, or which have an available English translation were considered. The search strategy included the following terms “spontaneous preterm birth” AND “biomarker” AND “blood”, including all relevant synonyms and alternate terms. Search terms and syntax were adjusted accordingly for each database, including the addition of relevant Medical Subject Headings (MeSH) where appropriate, and the searches were kept as similar as possible. No additional filters were applied to the searches. Complete search input for each database is described in S1 File.

### Study selection

Records retrieved were exported to a citation manager (Endnote Web). Duplicates were removed and the remaining articles were initially screened by title and abstract, those not relevant to the research question were removed. Remaining full-text articles were then assessed against the eligibility criteria. The reason for exclusion was noted for ineligible studies. Two investigators independently assessed the articles identified using the same search terms (KKH and EMW), and when a consensus could not be reached, a third reviewer acted as a tiebreaker (DMS).

### Quality assessment

The Newcastle-Ottawa Scale (NOS) scoring system was used to assess methodological quality and risk of bias for all eligible studies (S2 File). *Case-control studies* were assessed based on 1) adequate case definition for sPTB (in the case of multiple case groups, the paper was scored on only the case definition for sPTB), 2) representativeness of the cases, 3) selection of controls, 4) definition of controls with respect to history of sPTB, 5) comparability of cases and controls, 6) ascertainment of exposure/biomarker and 7) whether the same method of ascertainment for biomarker measurement was used in both cases and controls. *Cohort and cross-sectional studies* were assessed based on 1) cohort representativeness of the pregnant community, 2) assessment of outcome (in the case of multiple outcomes, the paper was scored on only the outcome definition for sPTB) and 3) adequacy of follow up. Methodological quality was independently rated by two reviewers, KKH and EMW. In the case of disagreement, ratings were discussed, and a consensus reached. Studies with total NOS scores below 50% were excluded from subsequent data extraction and synthesis.

### Data extraction

Data was extracted from eligible studies using a standardized template adapted from the Joanna Briggs Institute (JBI) data extraction form. The following data was collected from each study by a single reviewer (KH): objectives, participant characteristics, participant numbers, study setting, study year range, tissue (blood, serum or plasma), method of measurement of biomarker, timepoint of measurement, outcome of interest, country of origin, statistical test, effect measures as reported by odds ratios (OR), predictive value as reported by area under the receiver operator curve (AUC), biomarker levels in sPTB and term populations, results/direction, and comments pertaining to the heterogeneity of results. Missing or unclear information was marked ‘not stated’.

### Enrichment analysis of biomarkers

Top biomarkers were analyzed to identify common pathways or processes of interest. The goal of pathway analysis is to detect relevant groups of genes or proteins that are commonly associated with a biological function or process [11]. Genes or proteins are annotated based on current literature as they relate to a biological pathway, process, function, or localization which is condensed within databases. Pathway analysis can identify whether these annotations are enriched within a set of genes or proteins, in this case the set of biomarkers reported within the eligible studies. Any biomarker reported to be significantly associated with or predictive of sPTB in at least one study were analyzed with gProfiler for enrichment using the Gene Ontology (GO) database for cellular component, and the Kyoto Encyclopedia of Genes and Genomes (KEGG) pathway database.

## Results

### Summary of search results

**Fig 1:**
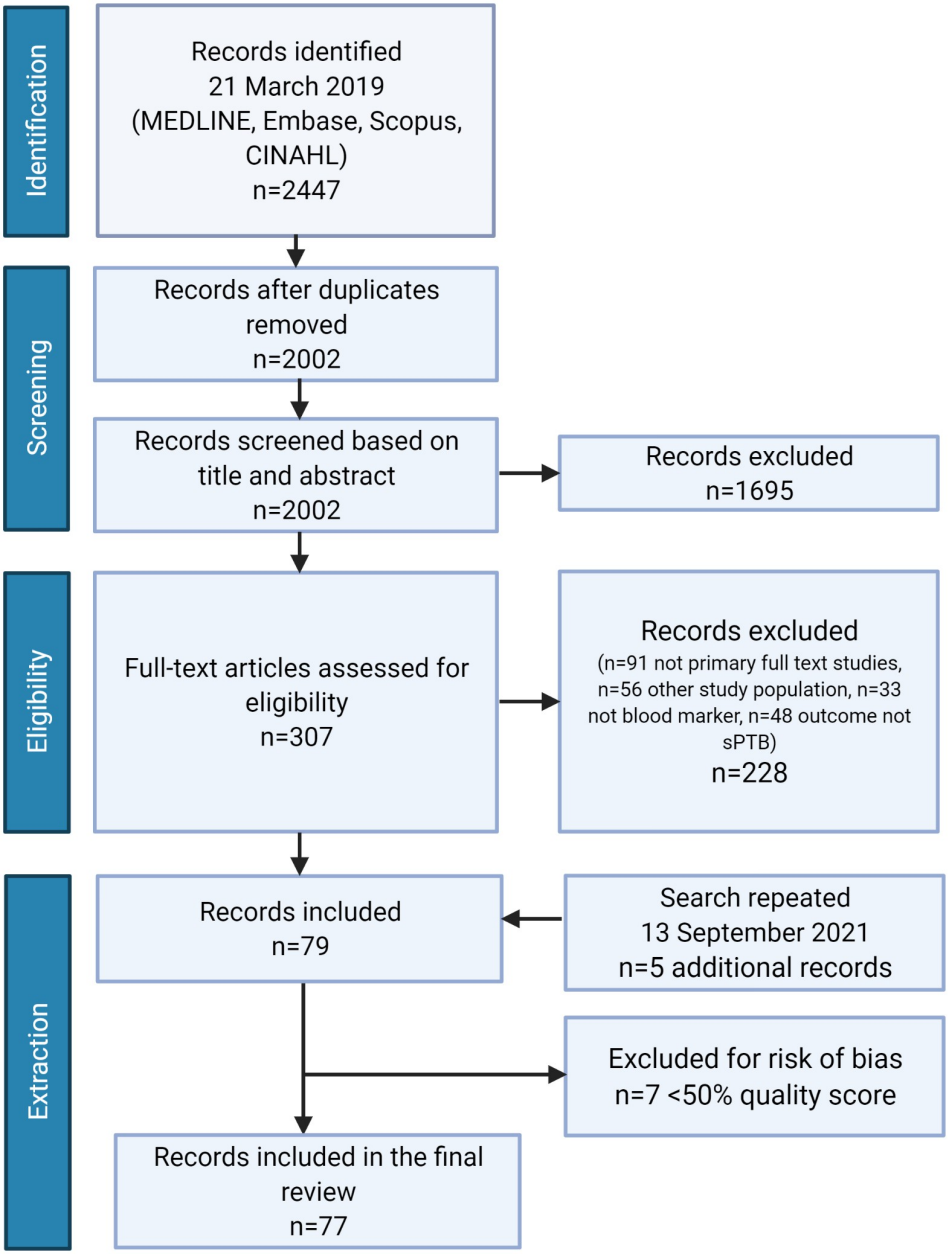
PRISMA flow diagram. Article identification, screening, and eligibility selection for systematic review of maternal blood markers associated with subsequent sPTB. Created with BioRender.com.

Search of the four databases retrieved 2002 non-duplicate records for screening. Screening of title and abstract identified 1695 records that were irrelevant to the research question and thus not further assessed for eligibility. A remaining 307 articles were assessed for eligibility using full-text records (Fig 1). The most common reason for record exclusion was study type, specifically review articles or conference abstracts (n = 91). Reference lists of review papers were reviewed, although no additional non-duplicate studies were identified. The remaining records were excluded by failing to meet the eligibility criteria for population (n = 23 studies measured biomarkers following the onset of labour symptoms, n = 33 did not meet criteria for healthy singleton pregnancies aged 18–35), exposure/marker (n = 1 amniotic fluid biomarker, n = 32 epigenetic/genetic biomarker), and outcome (n = 48 studies did not have sPTB as primary or secondary outcome). N = 66 and n = 76 studies identified by primary reviewer KKH and secondary reviewer EW, respectively, were cross-referenced by third reviewer DMS, leaving n = 79 studies included. The search was conducted again prior to submission to identify an additional n = 5 records, leaving a final total of n = 84 studies included for quality assessment.

### Methodological quality assessment

Quality assessment scores for cohort studies ranged from 3 to 7 out of a total possible 7 points (S2 File). Areas with the lowest scores among cohort studies were assessment of outcome and adequacy of follow up. Scores for case-control studies ranged from 3 to 9 out of a total possible 9 points. Areas with the lowest scores among case-control studies were adequate case definitions, representativeness of cases, and comparability of cases and controls. Of the 84 studies identified as eligible, n = 7 studies did not reach 50% on the NOS score and were excluded from subsequent data extraction, leaving a remaining n = 77 studies included in the final review (Table 1).

**Table 1:** Summary and quality assessment of studies included in review.

1° Author	No.	Year	Journal	Study Design	Quality Rating
Abdel Malek	[12]	2018	J Obstet Gynaecol	cohort	●●●●○○○
Akoto	[13]	2020	Sci Rep	cohort	●●●●○○○
Alleman	[14]	2013	Am J Obstet Gynaecol	case control	●●●●●●●○○
Ashrap	[15]	2020	Environ Int	cohort	●●●●●●○
Aung	[16]	2019	Sci Rep	case control	●●●●●○○○○
Bakalis	[17]	2012	J Matern Fetal Neonatal Med	case control	●●●●●●●○○
Bandoli	[18]	2018	J Perinatol	case control	●●●●●●○○○
Beta	[19]	2011	Fetal Diagn Ther	case control	●●●●●●●○○
Beta	[20]	2011	Prenat Diagn	case control	●●●●●●●○○
Beta	[21]	2012	J Matern Fetal Neonatal Med	case control	●●●●●●●○○
Bradford	[22]	2017	Clin Mass Spectrom	cohort	●●●●○○○
Bullen	[23]	2013	Reprod Sci	cohort	●●●●●●●
Cantonwine	[24]	2016	Am J Obstet Gynaecol	case control	●●●●●●●●●
Catov	[25]	2014	Am J Epidemiol	case control	●●●●●●○○○
Considine	[26]	2019	Metabolites	case control	●●●●●○○○○
Curry	[27]	2007	Acta Obstet Gynaecol Scand	case control	●●●●●●●○○
Curry	[28]	2009	Acta Obstet Gynaecol Scand	case control	●●●●●●●○○
Dhaifalah	[29]	2014	J Matern Fetal Neonatal Med	case control	●●●●●●○○○
El-Achi	[30]	2020	Fetal Diagn Ther	cohort	●●●●●●●
Esplin	[31]	2011	Am J Obstet Gynaecol	case control	●●●●●●○○○
Ezrin	[32]	2015	Am J Perinatol	case control	●●●●●●●●●
Ferguson	[33]	2014	Am J Reprod Immunol	case control	●●●●●●○○○
Goldenberg	[34]	2000	Am J Obstet Gynaecol	case control	●●●●●●●○○
Goldenberg	[35]	2001	Am J Obstet Gynaecol	case control	●●●●●○○○○
Gupta	[36]	2015	J Obstet Gynaecol	case control	●●●●●●●○○
Hackney	[37]	2010	Am J Obstet Gynaecol	case control	●●●●●●●○○
Heng	[38]	2016	PLoS ONE	case control	●●●●●●●●○
Huang	[39]	2019	Cytokine	case control	●●●●●○○○○
Huang	[40]	2020	Biomed J	cohort	●●●●●●○
Hvilsom	[41]	2002	Acta Obstet Gynaecol Scand	case control	●●●●●●●○○
Inan	[42]	2018	J Matern Fetal Neonatal Med	cohort	●●●●●○○
Jelliffe-Pawlowski	[43]	2010	Prenat Diagn	cohort	●●●●●●●
Jelliffe-Pawlowski	[44]	2013	Am J Obstet Gynaecol	case control	●●●●●●●●○
Jelliffe-Pawlowski	[45]	2015	Int J Obstet Gynaecol	cohort	●●●●●●●
Jelliffe-Pawlowski	[46]	2018	J Perinatol	case control	●●●●●●●●○
Kansu-Celik	[47]	2019	J Matern Fetal Neonatal Med	case control	●●●●●●●○○
Khambalia	[48]	2015	Brit J Nutr	cohort	●●●●●●○
Kirkegaard	[49]	2010	Prenat Diagn	cohort	●●●●●●○
Kirkegaard	[50]	2011	Prenat Diagn	cohort	●●●●●●○
Kwik	[51]	2003	Aust NZ J Obstet Gynaecol	cohort	●●●●○○○
Leung	[52]	1999	Brit J Obstet Gynaecol	cohort	●●●●●●○
Lynch	[53]	2016	Am J Obstet Gynaecol	case control	●●●●●○○○○
Ma	[54]	2020	J Clin Lab Anal	case control	●●●●●●●●○
Manuck	[55]	2021	Epigenomics	case control	●●●●●●●●●
McDonald	[56]	2015	PLoS ONE	cohort	●●●●●○○
McElrath	[57]	2019	Am J Obstet Gynaecol	case control	●●●●●●●●○
Mclean	[58]	1999	Am J Obstet Gynaecol	cohort	●●●●●●●
Ngo	[59]	2018	Science	cohort	●●●●●○○
Olsen SF	[60]	2018	EBioMedicine	case control	●●●●●●●○○
Olsen RN	[61]	2014	J Matern Fetal Neonatal Med	cohort	●●●●○○○
Parry	[62]	2014	Am J Obstet Gynaecol	case control	●●●●●●●●○
Paternoster	[63]	2002	Int J Obstet Gynaecol	cohort	●●●●○○○
Patil	[64]	2014	J Obstet Gynaecol India	cohort	●●●●○○○
Petersen	[65]	1992	Brit J Obstet Gynaecol	case control	●●●●●○○○○
Pihl	[66]	2009	Prenat Diagn	case control	●●●●●●●○○
Pihl	[67]	2009	Prenat Diagn	case control	●●●●●●●○○
Pitiphat	[68]	2005	Am J Epidemiol	case control	●●●●●●●●○
Poon	[69]	2009	Prenat Diagn	case control	●●●●●○○○○
Poon	[70]	2013	Fetal Diagn Ther	cohort	●●●●●○○
Ruiz	[71]	2002	Biol Res Nurs	cohort	●●●●●●○
Saade	[72]	2016	Am J Obstet Gynaecol	case control	●●●●●●●●●
Shin	[73]	2016	Taiwan J Obstet Gynaecol	case control	●●●●●●●○○
Sibai	[74]	2005	Am J Obstet Gynaecol	cohort	●●●●●○○
Smith	[75]	2007	Obstet Gynaecol	case control	●●●●●●○○○
Smith	[76]	2006	Int J Epidemiol	cohort	●●●●●○○
Soni	[77]	2018	J Matern Fetal Neonatal Med	case control	●●●●●●●○○
Spencer	[78]	2008	Ultrasound Obstet Gynaecol	cohort	●●●●○○○
Stegmann	[79]	2015	Fertil Steril	case control	●●●●●●●●○
Tarca	[80]	2021	Cell Rep	case control	●●●●●●●●○
Tripathi	[81]	2014	J Pregnancy	case control	●●●●●●●○○
Vogel	[82]	2006	Am J Obstet Gynaecol	cohort	●●●●○○○
Vogel	[83]	2007	J Reprod Immunol	cohort	●●●●○○○
Whitcomb	[84]	2009	J Women’s Health	case control	●●●●●●●○○
Winger	[85]	2020	PloS ONE	case control	●●●●●●○○○
Wommack	[86]	2018	PLoS ONE	case control	●●●●●●○○○
Zhou	[87]	2020	Reprod Sci	case control	●●●●●●●○○
Zhu	[88]	2018	Clin Chim Acta	cohort	●●●●●○○

Quality rating out of a maximum total 7 (cohort studies) or 9 (case control studies) points, as described by the quality assessment rubric (S2 File).

### Study characteristics

Data extraction was performed on the eligible n = 77 papers. Studies included 48 case-control designs and 29 cohort studies. Study details including participant number, country of origin, methods and results are outlined in S3 File. A total of n = 278 unique biomarkers were identified as significantly associated with or predictive of sPTB in at least one study (S4 File).

### Prediction models for preterm birth

Analysis methods from the 77 studies included, in order of complexity, univariate analysis comparing biomarker levels in sPTB pregnancies compared to term pregnancies (e.g., student’s t-test), binary classification (e.g., receiver operating characteristic curve analysis), multivariate models (e.g., linear or logistic regression), and machine learning models (e.g., random forest). Table 2 outlines the results from the 25 studies that conducted prediction of preterm birth using maternal blood biomarkers, as described by the sensitivity, specificity, and AUC of the classification. The AUC or area under the receiver operator curve is a performance metric for classification models, an AUC of 1.0 represents a perfect classifier, whereas an AUC of 0.5 represents random classification and thus is not a useful model. Top performing models included biomarkers identified through proteomic investigation, A2MG, HEMO, MBL2 [24], and ITIH4 [31], with an AUC of 0.89.

**Table 2:** Top prediction models for sPTB.

Paper	Biomarker(s)	GA	Analysis	Sensitivity	Specificity	AUC
Alleman 2013	maternal characteristics;	TM1	LogR	0.18	0.97	0.70
	TM1 total cholesterol;					
	dT total cholesterol;	TM2				
	TM2 AFP; INHBA					
Aung 2019	5-HETE; LTD4; LTC4-ME; 13-oxoODE; 5-oxoETE; 8-HETE; LTB4	23.1–28.9 wks	random forest	0.43	0.80	0.82(0.68–0.96)
Cantonwine 2016	A2MG; HEMO; MBL2	10–12 wks	LinR	0.80	0.83	0.89(0.83–0.95)
El Achi 2020	maternal characteristics; PAPPA	11–13 wks	LogR	na	na	0.67
Esplin 2011	ITIH4	24 and 28 wks	LogR	0.87	0.81	0.89(0.82–0.97)
Goldenberg 2000	CSF3	24 wks	LogR	0.54	0.79	na
		28 wks		0.37	0.95	
Heng 2016	*LMLN2; MIR3691; EFHD2; CST13P; ACAP2; ZNF324; SH3PXD2B; TBX21*; history of abortion and anemia	17–23 wks	LogR	0.65	0.88	0.84
		27–33 wks				
Huang 2019	INHBA	15–20 wks	ROC	na	na	0.57(0.64–0.51)
Inan 2018	PROK1	11–13 wks	ROC	0.77	0.52	0.69(0.59–0.78)
Jelliffe-Pawlowski 2010	AFP; hCG; uE3	15–20 wks	LogR	0.26	0.80	na
Jelliffe-Pawlowski 2013	PAPPA; AFP; INHBA	10–13 or 15–20 wks	LogR	na	na	na
Kansu Celik 2019	AGE	11–13 wks	ROC	0.92	0.74	0.82(0.91–0.93)
Leung 1999	CRH	15–20 wks	ROC	0.73	0.78	0.79
Ma 2020	neutrophil to lymphocyte ratio; hemoglobin; platelet distribution width	20–30 wks	LogR	0.89	0.41	na
Manuck 2021	*B2M; RUNX3; TLR4*	<28 wks	ROC	0.62	0.87	0.82(0.75–0.90)
McElrath 2019	F13A1; FBLN1; SERPING1; ITIH2; LCAT	10–12 wks	ROC	0.70	0.81	0.74(0.63–0.81)
Olsen RN 2014	uE3	15–22 wks	LogR	0.05	0.98	na
Saade 2016	IGFBP4; SHBG	17–28 wks	ROC	0.75	0.74	0.75
Shin 2016	IGFBP3	11–18 wks	LogR	1.00	0.70	0.79(0.66–0.89)
Smith 2006	AFP, hCG	Second trimester	logR	0.05	0.99	0.67(0.63–0.71)
Tarca 2021	50 proteins	27–33 wks	random forest	na	na	0.76(0.72–0.80)
Vogel 2006	relaxin	12–25 wks	LogR	0.45	0.19	0.64(0.48–0.79)
Vogel 2007	TNFa; cervical sIL-6Ra; short cervix	12–25 wks	LinR	0.96	0.95	na
Winger 2020	MIR181; MIR221; MIR33A; MIR6752; MIR1244; MIR148A; MIR1-1; MIR1267; MIR223; MIR199B; MIR133B; MIR144	6–12 wks	ROC	0.89	0.71	0.80(0.69–0.88)
Zhu 2018	MIF	<14 wks	LogR	0.79	0.51	0.71(0.64–0.77)

Top performing model from each paper which reported a prediction model for sPTB using maternal blood biomarkers, as measured by area under the receiver operating curve (AUC). GA: gestational age at sample collection. LogR: logistic regression. LinR: linear regression, ROC: receiver operator curve characteristic analysis.

### Enrichment analysis of biomarkers

Based on gene ontology analysis for cellular component, 47 cellular component GO terms were enriched within the dataset. Nine of the top ten enriched terms are nested within the ‘extracellular region’ GO term, indicating there is significant enrichment of biomarkers localized to the extracellular space. Enrichment analysis using the HPA database found significant enrichment of biomarkers originating from placental syncytiotrophoblast cell bodies (p-adj = 0.006). A total 53 KEGG pathways were significantly enriched within the list of biomarkers. Of these, 32 pathways within the KEGG class ‘Human Diseases’ were excluded, as they were deemed not relevant to the physiology of labour and preterm labour. The remaining 21 enriched pathways (Table 3) were classed as ‘Immune’, ‘Signal Transduction’, ‘Signaling molecules and interaction’ ‘Development and regeneration’ and ‘Cell growth and death’.

**Table 3:** Enriched pathways within the set of biomarkers.

Pathway	KEGG ID	KEGG class	p-adj	Ratio Total	Biomarkers
Complement and coagulation cascades	4610	Immune	2.07E-20	24/85	A2M; C1R; C3; C4A; C5; C8A; C9; CFB; CFH; F13A1; F13B; **F2**; F9; KLKB1; KNG1; MBL2; **PLAUR**; PLG; SERPINA1; SERPINC1; SERPINE1; SERPINF2; SERPING1; VTN
IL-17 signaling pathway	4657	Immune	5.61E-12	18/92	CCL11; CCL2; CCL7; CSF2; CSF3; CXCL5; CXCL8; HSP90AB1; IFNG; IL13; IL17A; IL17F; IL1B; IL4; IL5; **IL6**; MMP9; TNF
Intestinal immune network for IgA production	4672	Immune	0.001749	7/45	IL10; IL2; IL4; IL5; **IL6**; PIGR; TGFB1
Th1 and Th2 cell differentiation	4658	Immune	8.91E-07	13/89	IFNG; IL12A; IL13; IL2; IL4; IL4R; IL5; JAG1; NOTCH3; PPP3CA; PPP3R1; RUNX3; TBX21
Th17 cell differentiation	4659	Immune	6.95E-07	14/104	HSP90AB1; IFNG; IL17A; IL17F; IL1B; IL2; IL4; IL4R; **IL6**; IL6ST; PPP3CA; PPP3R1; TBX21; TGFB1
TNF signaling pathway	4668	Immune	2.11E-07	15/112	CCL2; CCL5; CSF1; CSF2; CXCL5; ICAM1; IFNB1; IL1B; **IL6**; JAG1; MAP2K4; MMP9; TNF; TNFRSF1A; TNFRSF1B
Cytokine-cytokine receptor interaction	4060	Signaling molecules and interaction	4.35E-21	39/293	CCL11; CCL2; CCL4; CCL5; CCL7; CSF1; CSF2; CSF3; CXCL5; CXCL8; CXCL9; CXCR3; FASLG; IFNB1; IFNG; IL10; IL10RA; IL12A; IL13; IL17A; IL17F; IL18; IL1B; IL1R2; IL2; IL4; IL4R; IL5; **IL6**; IL6ST; INHBA; LEP; NGF; ***PPBP***; TGFB1; TNF; TNFRSF1A; TNFRSF1B; TNFSF10
Toll-like receptor signaling pathway	4620	Immune	4.8E-06	13/102	CCL4; CCL5; CXCL8; CXCL9; IFNB1; IL12A; IL1B; **IL6**; MAP2K2; MAP2K4; **TLR2**; TLR4; TNF
Hematopoietic cell lineage	4640	Immune	1.78E-05	12/95	CSF1; CSF2; CSF3; **FCER2**; IL1B; IL1R2; IL4; IL4R; IL5; **IL6**; KITLG; TNF
Fc epsilon RI signaling pathway	4664	Immune	0.00322	8/67	CSF2; IL13; IL4; IL5; MAP2K2; MAP2K4; PDPK1; TNF
T cell receptor signaling pathway	4660	Immune	0.00032	11/103	CSF2; IFNG; IL10; IL2; IL4; IL5; MAP2K2; **PDPK1**; PPP3CA; PPP3R1; TNF
JAK-STAT signaling pathway	4630	Signal transduction	5.21E-06	16/162	CSF2; CSF3; IFNB1; IFNG; IL10; IL10RA; IL12A; IL13; IL2; IL4; IL4R; IL5; **IL6**; IL6ST; LEP; PDGFB
HIF-1 signaling pathway	4066	Signal transduction	0.003475	10/109	ANGPT1; ANGPT2; **CAMK2A**; FLT1; IFNG; **IL6**; MAP2K2; SERPINE1; TF; TLR4
Natural killer cell mediated cytotoxicity	4650	Immune	0.009761	10/123	CSF2; FASLG; ICAM1; IFNB1; IFNG; MAP2K2; PPP3CA; PPP3R1; TNF; TNFSF10
Osteoclast differentiation	4380	Development and regeneration	0.011175	10/125	CSF1; IFNB1; IFNG; IL1B; NCF1; PPP3CA; PPP3R1; TGFB1; TNF; TNFRSF1A
MAPK signaling pathway	4010	Signal transduction	4.99E-07	23/294	ANGPT1; ANGPT2; CSF1; DUSP1; FASLG; FGF2; FLT1; FLT4; HSPB1; IL1B; KDR; KITLG; MAP2K2; MAP2K4; MAPKAPK3; NGF; PDGFB; PGF; PPP3CA; PPP3R1; TGFB1; TNF; TNFRSF1A
PI3K-Akt signaling pathway	4151	Signal transduction	1.61E-07	26/353	ANGPT1; ANGPT2; CSF1; CSF3; FASLG; FGF2; FLT1; FLT4; FN1; HSP90AB1; IFNB1; IL2; IL4; IL4R; IL6; KDR; KITLG; MAP2K2; NGF; PDGFB; PDPK1; PGF; THBS1; **TLR2**; TLR4; VTN
Necroptosis	4217	Cell growth and death	0.018746	11/159	**CAMK2A**; FASLG; FTH1; HSP90AB1; IFNB1; IFNG; IL1B; TLR4; TNF; TNFRSF1A; TNFSF10
Rap1 signaling pathway	4015	Signal transduction	0.003747	14/210	ANGPT1; ANGPT2; CDH1; CSF1; FGF2; FLT1; FLT4; KDR; KITLG; MAP2K2; NGF; PDGFB; PGF; THBS1
Chemokine signaling pathway	4062	Immune	0.022609	12/190	CCL11; CCL2; CCL4; CCL5; CCL7; CXCL5; CXCL8; CXCL9; CXCR3; NCF1; ***PPBP***; PREX1
Ras signaling pathway	4014	Signal transduction	0.0385	13/231	ANGPT1; ANGPT2; CSF1; FASLG; FGF2; FLT1; FLT4; KDR; KITLG; MAP2K2; NGF; PDGFB; PGF

Enriched pathways within the set of biomarkers significantly associated with sPTB in at least one study. Of the total n = 278 biomarkers, n = 47 biomarkers were excluded from enrichment analysis as they did not correspond to a gene or protein (e.g., lipid, heavy metal, or cell type) and thus not compatible with pathway analysis. Ratio total represents the total number of biomarkers present within the dataset as a ratio of the total number of genes/proteins within the pathway. Protein biomarkers (regular black font): Gene expression biomarkers (underlined): Biomarkers altered at both the protein and gene expression levels (**bolded font**): Cell free DNA (cfDNA) biomarkers (***bold italic font***).

### Biomarkers from maternal serum screen tests

The most common biomarkers identified and analyzed were those measured during routine first and second trimester screening for aneuploidy, including pregnancy associated plasma protein A (PAPP-A), human chorionic gonadotropin (hCG), alpha fetoprotein (AFP), and estriol. Detailed odds ratios (OR) for maternal serum screen biomarkers summarized in S5 File.

#### PAPP-A

Low levels of PAPP-A were associated with sPTB (OR range 1.7–5.4) [20, 36, 44, 45, 49–51, 64, 78, 89], though results were mixed [14, 42, 66, 69, 70].

#### hCG

Evidence suggesting an association between maternal levels of hCG and sPTB is inconclusive [14, 20, 44, 50], though some studies suggest that low levels of hCG were associated with increased risk of sPTB (OR range 0.8–2.0) [43, 45, 49, 78] and that high levels of hCG independently decreased risk of sPTB [77].

#### AFP

High serum AFP was significantly associated with sPTB (OR range 1.9–8.3) [14, 19, 35, 43–45, 58, 90] (OR range 1.9–8.3), though one study showed no significant difference [81].

#### Estriol

Studies on serum estriol were split, while two studies showed a significant association between high estriol and sPTB [43, 61], others found no association [14, 44, 45].

### Endocrine markers

#### CRH

Elevated levels of corticotropin releasing hormone (CRH) were significantly associated with increased risk of sPTB [52, 71, 74]. Others reported elevated odds of sPTB with elevated levels of CRH, though the findings were not statistically significant [35, 58] (S5 File).

#### Cortisol

Serum cortisol levels were not associated with gestational age at delivery [18], nor was elevated cortisol associated with increased odds of sPTB [35].

#### AMH

Second trimester levels of anti-müllerian hormone (AMH) were not associated with sPTB: however, stable or rising levels of AMH in early pregnancy were associated with sPTB, but only in those with high levels of serum AFP [79].

### Lipids and biomarkers associated with lipid pathways

#### Cholesterol

Total cholesterol (TC) at the first trimester, as well as change in TC from the first to second trimesters, but not high density lipoproteins (HDL), low density lipoproteins (LDL) or triglyceride levels, improved prediction of sPTB compared to sPTB history alone, and performed similarly in those that did not have history of sPTB [14].

#### NEFAs

High maternal blood levels of non-esterified or “free” fatty acids (NEFA) (>0.33mmol/L) was associated with 2.02 (confidence interval or CI 1.13–3.48) increased odds of subsequent sPTB compared to low NEFA levels (<0.19mmol/L) [25].

#### Eicosanoids and other lipid markers

Biomarkers in the lipoxygenase, epoxygenase and cyclooxygenase pathways were investigated as potential biomarkers of sPTB. Lipid biomarkers alone performed similarly (AUC 0.79 CI 0.62–0.96) than a combined panel of inflammatory, oxidative stress and lipid biomarkers (AUC 0.79 CI 0.61–0.98) in distinguishing sPTB [16] from term births. Low levels of fatty acids eicosapentanaeoic acid (EPA) and docosahexaenoic acid (DHA) in the first and second trimesters was associated with 10-times increased risk of sPTB [60].

### Heterogeneity of sPTB

#### Ethnicity-specific biomarkers

The most common country of study origin was the United States of America, with majority non-Hispanic Caucasian or Black populations (S3 File). Corticotropin releasing hormone (CRH) was associated with sPTB in a majority ethnic Chinese population [52] and majority non-Hispanic Caucasian population [71], but not in a majority Black population [74]. Serum ferritin was associated with sPTB in Indian and Egyptian populations [12, 81], though results are mixed for Caucasian and Black populations in American and European countries [21, 31, 35, 63], which may suggest socio-demographic and ethnicity specific interactions within this biomarker. There is some suggestion that cortisol is associated with sPTB in Caucasian, but not Black populations [18, 35].

#### Parity

The predictive value of alpha-fetoprotein (AFP) and multi-marker models was higher in parous populations as compared to nulliparous [14, 19, 57], indicating that these two populations may have distinct physiology of sPTB and may require distinct approaches to prediction. Other biomarkers from maternal serum screen tests, pregnancy associated plasma protein (PAPP-A) and human chorionic gonadotropin (B-hCG) were similarly more associated with sPTB in parous populations with previous sPTB as compared to nulliparous populations [20, 77].

#### BMI

Inflammatory cytokines and CRP are more strongly associated with sPTB in populations with high BMI [23, 39], and that prediction using inflammatory biomarkers may be distinct in underweight and obese populations [28, 72].

#### Fetal sex-specific biomarkers

There is some suggestion that there are no fetal sex-specific differences in biomarker associations with sPTB [15, 58, 79]. However, one 2010 study found that low pregnancy associated plasma protein (PAPP-A) levels were more strongly associated with sPTB in pregnancies carrying a female fetus as compared to male [49].

### Gestational age considerations

Inflammatory biomarkers including cytokines and serum ferritin, biomarkers from maternal serum screen tests and CRH are more strongly associated with very early sPTB (<32 weeks gestation) as compared to moderate-late sPTB [34, 35, 45, 46, 48, 52, 76]. On the other hand, gene expression markers, and protein markers pro-MBP and SP1 may be more associated with overall sPTB (<37 weeks gestation) as compared to early [55, 66, 67], while estriol is more strongly associated with moderate sPTB (32–34 weeks) [61]. A 2015 study of Tanzanian women found distinct angiogenic biomarkers for each subtype of sPTB prematurity, indicating distinct physiologies across subtypes [91].

Of the 77 studies included in this review, only 10 reported biomarker measurements obtained at more than one time point. Three studies on transcript markers found that change in transcript levels was more predictive than absolute values [38, 80, 87]. On the other hand, Parry et al. [62], showed that change in proteomic markers was not highly associated with sPTB. Five additional studies did not investigate change in biomarker levels, but performed statistical analysis at each time point separately [15, 26, 33, 34, 37], while Esplin et al., [31] measured markers in separate cohorts for each time point. This limits our understanding of how biomarker levels change throughout gestation, which may be an important indicator for risk of sPTB. Inflammatory cytokines showed no difference in association when measured at different timepoints in gestation, except for IL10, which was most strongly associated with sPTB when measured after 22 weeks gestation [33, 34]. A 2010 study found that thrombin-antithrombin complexes (TAT) were more strongly associated with sPTB when measured later in gestation (28 weeks) as compared to earlier (24 weeks) [37]. On the other hand, a 2011 study found the free B-hCG was more strongly associated with sPTB with earlier sampling, but the same difference was not true of PAPP-A [50]. One study compared the predictive value at multiple time points and found that among a range of time points between 17–28 weeks, samples collected at 19–21 weeks were ideal for biomarker discovery [72].

## Discussion

### Main findings

Analysis of the seventy-seven papers identified via this systematic review suggest that there is no clear single biomarker or set of biomarkers in the current existing literature for the prediction of sPTB. Low levels of PAPP-A and elevated levels of AFP are associated with increased risk of sPTB; however, these results may be biased due to secondary use of data and incomplete datasets. Consistent study design, which would facilitate systematic meta-analyses of these studies, would be necessary to validate these results before confirming any clinical utility of these markers. Further, although inflammation has long been associated with labour and sPTB, studies investigating the association between inflammatory biomarkers and sPTB are inconclusive. G-CSF was found to have the strongest and most consistent association with sPTB, but there is insufficient evidence to support an association with other biomarkers associated with systemic inflammation such as IL-1B, TNFa, CRP, IL-6, IL-2, IFNy, IL-10 and serum ferritin. However, the emergence of omics technologies has identified biomarkers and pathways of interest that may identify novel avenues for prediction. It is likely that no single biomarker will be predictive of sPTB but high-throughput technologies for biomarker discovery, improved feature selection, and integration with other known risk factors such as cervical length and history of sPTB may provide a set of biomarkers with clinical utility for the prediction of sPTB.

### The preterm birth phenotype

This systematic review was the first, to our knowledge, to systematically review the existing literature on maternal blood markers, collected before any signs of labour, that are predictive of a spontaneous preterm birth. From our initial search, forty-eight primary studies were excluded for not defining preterm birth following spontaneous labour or rupture of membranes as their primary or secondary outcome(s). Outcomes were typically preterm delivery (<37 weeks), with no reference to primary records that would differentiate the spontaneous PTBs from those that follow physician initiation, otherwise referred to as medically indicated PTBs. These medically indicated births and those arising spontaneously are not likely to have a shared etiology, and thus identifying common biomarkers for all PTB subtypes is unlikely; this was the basis of our justification to exclude these papers from review.

A limitation of the studies reviewed is the potential for misclassification bias due to poor outcome definitions. Most studies did not provide reference to primary records that define the sPTB outcome. With respect to gestational age, misclassification bias is most likely for those preterm cases that occur near term (~36 weeks), depending on the method by which gestational age was determined (last menstrual period, ultrasound etc.). With respect to other obstetric outcomes, most studies failed to provide repeatable protocols on how sPTBs were differentiated from those that were medically indicated, and the extent of missing data may have affected the risk of bias within the reported results.

While preterm birth is often regarded as a single outcome in clinical practice, the preterm birth phenotype has multiple and complex etiologies. There is no recognized system for grouping preterm birth phenotypes, and the known etiologies include pre-eclampsia, multiple births, infection, fetal growth restriction, fetal distress, decidual hemorrhage and placental dysfunction [92]. However, 30% of preterm births, a greater proportion than any other etiology, are not associated with known maternal, fetal, or placental conditions, but exhibit spontaneous contractions or rupture of membranes [93]. Increased fetal mortality and morbidity is not only associated with lower gestational age at delivery, but also with the different patterns of PTB etiology [94]. For example, it is likely that extreme PTBs (<28 weeks gestation) have a different etiology than those occurring near term. Larger studies that allow for a high degree of stratification, such as by gestational age (extreme PTB <28 weeks, very PTB 28–32 weeks, or moderate-late PTB 32–37 weeks), and by potential etiology may identify novel biomarkers unique to each phenotype and may mediate the effects of the heterogeneity. Biomarker discovery for early detection of highly heterogenous outcomes requires at least 2-fold larger sample sizes and different statistical considerations than if the outcome were homogenous, which must be put into consideration when performing power calculations during study design [95].

### Considerations for sample collection

Our results suggest that change in biomarkers over multiple measurements throughout gestation is more strongly associated with sPTB than single timepoint measurements, though this may be dependent on the type of biomarker measured. Not only is there evidence to suggest that multiple measurements are beneficial for biomarker discovery and predicting sPTB, understanding the dynamics of molecular changes throughout gestation would also provide greater insight to the mechanisms of sPTB. Sample collection in large cohorts is time consuming and costly, not excluding the time for additional analyses, which is further exacerbated by multiple measurements. While there is evidence to suggest that two measurements are beneficial for predicting sPTB, there is insufficient evidence to suggest that more than two would provide any added benefit. Further, our results demonstrate that biomarkers measured in the second and third trimesters are more predictive of sPTB than those measured earlier, though this likely depends on the biomarker. Results of a chi-squared analysis of markers measured in both serum and plasma found no statistical difference in the likelihood of reporting an association with sPTB (p>0.05). There is insufficient evidence to suggest an advantage to collecting plasmas over serums, and as serums are often collected as part of routine antenatal screening, this may be a more convenient biological tissue to use for biomarker discovery. However, a direct comparison of biomarkers measured in both serums and plasmas would be necessary to investigate the use of either biofluid.

### The ‘Big Data’ era

High throughput technologies such as microarrays, next generation sequencing and mass spectrometry now allow for the generation of large datasets including proteomic, metabolomic, genomic and transcriptomic information. These technologies, along with subsequent bioinformatic analyses, allows for an unprecedented opportunity to identify novel biomarkers [96]. Investigations into maternal biomarkers for sPTB are often limited by the currently limited understanding of the molecular mechanisms of labour. For example, though there is an ongoing hypothesis that labour is an inflammatory process [97], we show here that biomarkers of systemic inflammation such as IL-1B and CRP are not associated with sPTB. The process of labour may certainly involve inflammatory mediators but is likely much more complex than a simple switching on or off, of inflammation. These large omics-datasets, while identifying novel markers, also allow for investigation into global molecular changes, providing additional insight into novel pathways and molecules that may be involved in the mechanism of preterm birth. In particular, the field of metabolomics is largely under studied with respect to prediction of sPTB, only one study met the inclusion criteria for this review [26]. The field of parturition requires further investigation using these high-throughput technologies to facilitate identification of better biomarkers with clinical utility for prediction of preterm birth.

With the advent of such high-throughput technologies and increasingly high-dimensional datasets comes the need for more robust forms of data analysis and mining. Machine learning and other complex data analysis methods are particularly well suited for high-dimensional and complex data as they do not generally require the data to adhere to any *a priori* assumptions about linearity of distribution [98]. These techniques can be used to identify subtle, complex and interactive patterns within datasets which can subsequently be leveraged for prediction and discovery of phenotypes such as sPTB [96]. A major limitation to machine learning is the risk of overfitting the data, which is especially problematic when the number of features far exceeds the number of observations, as is common in -omics datasets in health sciences. This drives the impetus for dimensionality reduction and/or feature selection, which can reduce the risk of overfitting, reduce computational resources required for analysis and, in the case of supervised feature selection, can identify the most important features for prediction. A recent study identified intra- and extra-uterine factors most informative of sPTB to estimate risk of sPTB using a random forest classifier with high predictive performance (AUC 0.81) [99]. The etiology of sPTB is multi-factorial, and is likely driven by more complex, subtle interactions which machine learning approaches, along with feature selection for identifying informative features, is well suited to detect.

### Limitations

This review was not preregistered. Pre-registering systematic reviews can reduce potential for bias, increase transparency and avoid unintended duplication of reviews [100]. Though there have been recently published systematic reviews on the topic of biomarkers of PTB in 2017 and 2018 [101, 102], there has not, to our knowledge, been a systematic review of biomarkers specific to the maternal blood compartment prior to the onset of labour. Further, studies published within the last 5 years since their publication, particularly those using high-throughput proteomics and transcriptomic techniques have identified novel biomarkers of sPTB, highlighting the value of revisiting the literature. A 2002 study of 47 systematic reviews found that 91.5% of Cochrane reviews contained major changes to their methods and selected outcomes as compared to their pre-registered protocols [103], suggesting a potential risk of bias in the reported outcomes.

Another limitation of this study is that we did not collect grey literature (e.g. unpublished work). Non reporting biases contribute to missing results in this systematic review, likely skewing associations with sPTB more positively. Negative results, in other words, biomarkers that have been found to not be predictive of sPTB, are less likely to be published due to high p value, the magnitude or direction of the results. Biomarkers that did not meet thresholds for statistical significance in preliminary/bivariate or univariate analysis were often excluded from downstream analyses, or the data was not shown, suggesting that there was further under-reporting of results due to insufficient p-values. Further, support for why the biomarkers of interest were selected in each study was often missing, not thorough, or not compelling. Studies that prospectively register their protocols are more likely to report negative results [104]; however, observational studies, which make up the majority of studies included in this review, are not typically prospectively registered, limiting options to reduce bias due to missing results. Lastly, we did not perform a meta-analysis, primarily due to inconsistent study designs which preclude options for meta-analyses but also that a meta-analysis of biomarkers assessed in the literature would not be particularly useful as it would be so heavily biased by the authors’ choice of biomarker—apart from, perhaps, -omics studies. Meta-analysis of the -omics studies was limited by highly inconsistent study designs and thus was not conducted.

## Conclusions

Currently, there is no known clear single biomarker, or set of biomarkers, for the prediction of spontaneous preterm birth. This review highlights that current biomarker discovery techniques are largely limited by the heterogenous nature of preterm birth and an incomplete understanding of the mechanisms that drive this process. Omics-style studies with more robust feature selection and analytical approaches provide a promising avenue for the identification of novel biomarkers. Larger studies with adequate power and more consistent study design, namely, clearly defined outcomes that consider the heterogeneity and subtypes of PTB, are needed to identify a set of biomarkers predictive of sPTB.

## Supporting information

S1 File Database search strategy. (DOCX)

S2 File Methodological quality assessment rubrics. (DOCX)

S3 File Data extraction. (PDF)

S4 File Biomarkers of sPTB. (DOCX)

S5 File Odds ratios for top biomarkers. (DOCX)

S1 Checklist PRISMA checklist. (DOCX)

S2 Checklist PRISMA abstract checklist. (DOCX)
