# Supplementary material for: Is there a maternal blood biomarker that can predict spontaneous preterm birth prior to labour onset? A systematic review
Source: PLoS One. 2022 Apr 4;17(4):e0265853. doi: 10.1371/journal.pone.0265853 (PMC8979439; doi:10.1371/journal.pone.0265853)
Supplement: S1 File — (DOCX) [file pone.0265853.s001.docx]

**S1 File. Database search strategy**

**MEDLINE and EMBASE search strategy**

| **Concept** | **MeSH Term** | **Other terms** |
| --- | --- | --- |
| Preterm labour | Obstetric Labor, Premature/ | Preterm labor  Preterm labour  Pre-term labor  Pre-term labour  Premature labor  Premature labour  Pre-mature labor  Pre-mature labour |
| Biomarker | Biomarkers/ | Biomarker*  Biologic* marker*  Clinical marker *  Immune marker*  Immunologic* marker*  Serum marker *  Sero* marker* |
| Expression | Gene Expression/  Transcription, Genetic/  Transcriptome/ | Gene express*  Gene transcript*  Genes express*  Genes transcript*  Genetic express*  Genetic transcript*  Transcri* |
| Maternal peripheral blood |  | Plasma adj2 maternal  Serum adj2 maternal  Serologic* adj2 maternal  Blood adj2 maternal |

Search strategy: (Biomarker OR (Maternal peripheral blood AND Expression) OR (Biomarker AND Maternal peripheral blood) OR (Expression AND Biomarker And Maternal peripheral blood)) AND Preterm labour

**Scopus search strategy**

| **Concept** | **Field** | **Other terms** |
| --- | --- | --- |
| Preterm Labour | Title-abs-key | Preterm labor  Preterm labour  Pre-term labor  Pre-term labour  Premature labor  Premature labour  Pre-mature labor  Pre-mature labour |
| Biomarker | Title-abs-key | Biologic* marker*  Clinical marker*  Gene marker*  Genes marker*  Genetic* marker*  Immune marker*  Immunologic* marker*  Serum marker*  Sero* marker*  Biomarker*  Gene express*  Genes express*  Genetic* express*  Gene transcript*  Genes transcript*  Genetic* transcript*  Transcri* |
| Maternal peripheral blood | Title-abs-key | Plasma w/2 maternal  Serum w/2 maternal  Serologic w/2 maternal  Blood w/2 maternal  Maternal w/2 whole w/2 blood |
| Prediction | Title-abs-key | Predictive  Predict*  Screen*  Prenatal w/3 screen*  Prenatal w/2 test*  Pre-natal w/3 screen*  Pre-natal w/3 test*  Maternal w/3 screen*  Maternal w/3 test* |

Search strategy: Preterm labour AND Biomarker AND Maternal peripheral blood AND Prediction

**CINAHL search strategy**

| **Concept** | **Other terms** |
| --- | --- |
| Preterm labour | Preterm labor  Preterm labour  Pre-term labor  Pre-term labour  Premature labor  Premature labour  Pre-mature labor  Pre-mature labour |
| Biomarker | Biomarker*  Biologic* marker*  Clinical marker *  Immune marker*  Immunologic* marker*  Serum marker *  Sero* marker* |
| Expression | Gene express*  Gene transcript*  Genes express*  Genes transcript*  Genetic express*  Genetic transcript*  Transcri* |
| Maternal peripheral blood | Plasma N2 maternal  Serum N2 maternal  Serologic* N2 maternal  Blood N2 maternal |

Search strategy: (Biomarker OR (Maternal peripheral blood AND Expression) OR (Biomarker AND Maternal peripheral blood) OR (Expression AND Biomarker And Maternal peripheral blood)) AND Preterm labour
