# Supplementary material for: Is there a maternal blood biomarker that can predict spontaneous preterm birth prior to labour onset? A systematic review
Source: PLoS One. 2022 Apr 4;17(4):e0265853. doi: 10.1371/journal.pone.0265853 (PMC8979439; doi:10.1371/journal.pone.0265853)
Supplement: S2 File — (DOCX) [file pone.0265853.s002.docx]

**S2 File. Methodological quality assessment rubrics**

**Table 1. Methodological quality assessment rubric for case control studies**

|  | **2pt** | **1pt** | **0pt** |
| --- | --- | --- | --- |
| **Is the case definition adequate?** | outcome (primary or secondary) is defined as preterm (<37 weeks or earlier) birth occurring after spontaneous onset of labour, with or without rupture of membranes. Independently validated with reference to primary medical/hospital records | outcome (primary or secondary) is defined as preterm (<37 weeks or earlier) birth occurring after spontaneous onset of labour, with or without rupture of membranes. Self-reported or record linkage with no reference to primary record | no description (*note: studies which did not describe outcome as sPTB were excluded) |
| **Representativeness of the cases** |  | study includes consecutive or obviously representative series of cases: all cases with outcome of interest over a defined period of time, all cases in a defined catchment area, all cases in a defined hospital or clinic, group of hospitals, health maintenance organisation, or an appropriate sample of those cases (e.g. random sample) | potential for selection biases (did not meet requirements in cell D3) or not stated |
| **Selection of controls** |  | Controls used in the study is derived from the same population as the cases and essentially would have been cases had the outcome been present (same community) | no description, controls not from same community |
| **Definition of controls** |  | if cases are first occurrence of outcome (i.e. no history of sPTB), then it must be explicitly stated that controls have no history of sPTB. If cases have new (not necessarily first) occurrence of sPTB, then controls with previous occurrences of sPTB should not be excluded | no mention of history of sPTB |
| **Comparability of cases and controls on the basis of the design or analysis** |  | Cases and controls are matched in the design and/or confounders are adjusted for in the analysis (factors appropriate will vary based on biomarkers) | Cases and controls are not matched and/or confounders are not adjusted for in the analysis. Compatibility of cases and controls show evidence of bias. |
| **Ascertainment of exposure** | biomarkers are measured in blood using methods that are sufficiently described so as to be repeatable, researcher blinded to case/control status | methods insufficiently described so as to be repeatable, researcher not blinded to case/control status | no description (insufficient) |
| **Same method of ascertainment for cases and controls** |  | biomarkers were measured in blood using identical methods for cases and controls | biomarkers were measured in blood using different methods in cases and controls |

Adapted from the Newcastle Ottawa Scale

**Table 2. Methodological quality assessment rubric for cohort studies**

|  | 2pt | 1pt | 0pt |
| --- | --- | --- | --- |
| **Representativeness of the cohort** | truly or somewhat representative of the average pregnant community | selected group (e.g. volunteers) | no description of the derivation of the cohort |
| **Ascertainment of exposure** | biomarkers are measured in blood using methods that are sufficiently described so as to be repeatable, researcher blinded to outcome status (if retrospective) | methods insufficiently described so as to be repeatable, researcher not blinded to outcome status (if retrospective) | no description (insufficient) |
| **Assessment of outcome** | outcome (primary or secondary) is defined as preterm (<37 weeks or earlier) birth occurring after spontaneous onset of labour, with or without rupture of membranes. Independently validated with reference to primary medical/hospital records | outcome (primary or secondary) is defined as preterm (<37 weeks or earlier) birth occurring after spontaneous onset of labour, with or without rupture of membranes. Self-reported or record linkage with no reference to primary record | no description (*note: studies which did not describe outcome as sPTB were excluded) |
| **Adequacy of follow up of cohorts** |  | complete follow-up, all subjects accounted for, or subjects lost to follow up unlikely to introduce bias (small number or a description provided of those lost) | subjects lost presents likely introduction of bias and/or no description provided for those lost |

Adapted from the Newcastle Ottawa Scale

**Table 3. Methodological quality assessment of included case control studies**

| **Study** | **Case Definition** | **Representativeness of the cases** | **Selection of controls** | **Definition of controls** | **Comparability of cases and controls** | **Ascertainment of exposure** | **Same method** | **Total Score** |
| --- | --- | --- | --- | --- | --- | --- | --- | --- |
| Alleman 2013 | 1 | 1 | 1 | 1 | 1 | 1 | 1 | 7 |
| Aung 2019 | 0 | 1 | 1 | 0 | 1 | 1 | 1 | 5 |
| Bakalis 2012 | 1 | 1 | 1 | 1 | 1 | 1 | 1 | 7 |
| Bandoli 2018 | 1 | 0 | 1 | 0 | 1 | 2 | 1 | 6 |
| Beta 2011 (Prenat Diagn) | 1 | 1 | 1 | 1 | 1 | 1 | 1 | 7 |
| Beta 2011 (Fetal Diagn Ther) | 1 | 1 | 1 | 1 | 1 | 1 | 1 | 7 |
| Beta 2012 | 1 | 1 | 1 | 1 | 1 | 1 | 1 | 7 |
| Cantonwine 2016 | 2 | 1 | 1 | 1 | 1 | 2 | 1 | 9 |
| Catov 2014 | 1 | 1 | 1 | 0 | 1 | 1 | 1 | 6 |
| Considine 2019 | 0 | 1 | 1 | 0 | 1 | 1 | 1 | 5 |
| Curry 2007 | 1 | 1 | 1 | 1 | 1 | 1 | 1 | 7 |
| Curry 2009 | 1 | 0 | 1 | 1 | 1 | 2 | 1 | 7 |
| D'Silva 2018 | 0 | 0 | 0 | 0 | 1 | 1 | 1 | 3* |
| D'Silva 2020 | 0 | 0 | 1 | 0 | 1 | 1 | 1 | 4* |
| Dhaifalah 2014 | 1 | 1 | 1 | 1 | 0 | 1 | 1 | 6 |
| Esplin 2011 | 1 | 1 | 1 | 0 | 0 | 2 | 1 | 6 |
| Ezrin 2015 | 2 | 1 | 1 | 1 | 1 | 2 | 1 | 9 |
| Ferguson 2014 | 1 | 1 | 1 | 0 | 1 | 1 | 1 | 6 |
| Goldenberg 2000 | 1 | 0 | 1 | 1 | 1 | 2 | 1 | 7 |
| Goldenberg 2001 | 0 | 1 | 1 | 1 | 1 | 0 | 1 | 5 |
| Gupta 2015 | 1 | 1 | 1 | 0 | 1 | 2 | 1 | 7 |
| Hackney 2010 | 1 | 1 | 1 | 1 | 1 | 1 | 1 | 7 |
| Heng 2016 | 2 | 1 | 1 | 1 | 1 | 1 | 1 | 8 |
| Huang 2019 | 0 | 1 | 1 | 0 | 1 | 1 | 1 | 5 |
| Hvilsom 2002 | 1 | 1 | 1 | 1 | 1 | 1 | 1 | 7 |
| Jelliffe Pawlowski 2013 | 2 | 1 | 1 | 1 | 1 | 1 | 1 | 8 |
| Jelliffe Pawlowski 2018 | 2 | 1 | 1 | 1 | 1 | 1 | 1 | 8 |
| Kansu Celik 2019 | 1 | 1 | 1 | 1 | 1 | 1 | 1 | 7 |
| Lynch 2016 | 1 | 1 | 1 | 0 | 0 | 1 | 1 | 5 |
| Ma 2020 | 2 | 1 | 1 | 0 | 1 | 2 | 1 | 8 |
| Manuck 2021 | 2 | 1 | 1 | 1 | 1 | 2 | 1 | 9 |
| McElrath 2019 | 1 | 1 | 1 | 1 | 1 | 2 | 1 | 8 |
| Movahedi 2012 | 0 | 1 | 1 | 0 | 0 | 0 | 1 | 3* |
| Navolan 2016 | 1 | 0 | 0 | 0 | 0 | 1 | 1 | 3* |
| Olsen SF 2018 | 2 | 0 | 1 | 0 | 1 | 2 | 1 | 7 |
| Parry 2014 | 1 | 1 | 1 | 1 | 1 | 2 | 1 | 8 |
| Peterson 1992 | 0 | 1 | 1 | 1 | 0 | 1 | 1 | 5 |
| Pihl 2009 | 2 | 1 | 1 | 0 | 1 | 1 | 1 | 7 |
| Pihl 2009 | 2 | 1 | 1 | 0 | 1 | 1 | 1 | 7 |
| Pitiphat | 1 | 1 | 1 | 1 | 1 | 2 | 1 | 8 |
| Poon 2009 | 0 | 0 | 1 | 1 | 1 | 1 | 1 | 5 |
| Saade 2016 | 2 | 1 | 1 | 1 | 1 | 2 | 1 | 9 |
| Shin 2016 | 0 | 1 | 1 | 1 | 1 | 2 | 1 | 7 |
| Smith 2007 | 1 | 1 | 1 | 0 | 1 | 1 | 1 | 6 |
| Soni 2018 | 1 | 1 | 1 | 1 | 1 | 1 | 1 | 7 |
| Stegmann 2015 | 1 | 1 | 1 | 1 | 1 | 2 | 1 | 8 |
| Tarca 2021 | 1 | 1 | 1 | 1 | 1 | 2 | 1 | 8 |
| Tripathi 2014 | 0 | 1 | 1 | 1 | 1 | 2 | 1 | 7 |
| Truong 2017 | 0 | 0 | 1 | 0 | 0 | 1 | 1 | 3* |
| Whitcomb 2009 | 1 | 1 | 1 | 1 | 1 | 1 | 1 | 7 |
| Winger 2020 | 1 | 0 | 1 | 1 | 0 | 2 | 1 | 6 |
| Wommack 2018 | 1 | 0 | 1 | 1 | 1 | 1 | 1 | 6 |
| Zhou 2020 | 2 | 1 | 1 | 0 | 1 | 1 | 1 | 7 |
| *Excluded from data extraction for high risk of bias | | |  |  |  |  |  |  |
|  |  |  |  |  |  |  |  |  |

**Table 4. Methodological quality assessment of included cohort studies**

| **Study** | **Representativeness of the cohort** | **Ascertainment of exposure** | **Assessment of outcome** | **Adequacy of follow up** | **Total Score** |
| --- | --- | --- | --- | --- | --- |
| Abdel Malek 2018 | 2 | 1 | 0 | 1 | 4 |
| Akoto 2020 | 2 | 1 | 1 | 0 | 4 |
| Ashrap 2020 | 2 | 2 | 2 | 0 | 6 |
| Bradford 2017 | 2 | 2 | 0 | 0 | 4 |
| Bullen 2013 | 2 | 2 | 2 | 1 | 7 |
| El-Achi 2020 | 2 | 2 | 2 | 1 | 7 |
| Huang 2020 | 2 | 1 | 2 | 1 | 6 |
| Inan 2017 | 2 | 1 | 1 | 1 | 5 |
| Jelliffe Pawlowski 2010 | 2 | 2 | 2 | 1 | 7 |
| Jelliffe Pawlowski 2015 | 2 | 2 | 2 | 1 | 7 |
| Khambalia 2015 | 2 | 1 | 2 | 1 | 6 |
| Kirkegaard 2010 | 2 | 2 | 2 | 0 | 6 |
| Kirkegaard 2011 | 2 | 2 | 2 | 0 | 6 |
| Kwik 2003 | 2 | 1 | 0 | 1 | 4 |
| Leung 1999 | 2 | 2 | 1 | 1 | 6 |
| Mcdonald 2015 | 2 | 2 | 0 | 1 | 5 |
| Mclean 1999 | 2 | 2 | 2 | 1 | 7 |
| Ngo 2018 | 2 | 1 | 1 | 1 | 5 |
| Olsen RN 2014 | 2 | 1 | 1 | 0 | 4 |
| Paternoster 2002 | 2 | 1 | 0 | 1 | 4 |
| Patil 2014 | 2 | 1 | 0 | 1 | 4 |
| Poon 2013 | 2 | 1 | 2 | 0 | 5 |
| Pummarra 2016 | 2 | 1 | 0 | 0 | 3* |
| Ruiz 2002 | 2 | 1 | 2 | 1 | 6 |
| Sibai 2005 | 2 | 2 | 0 | 1 | 5 |
| Smith 2006 | 2 | 1 | 2 | 0 | 5 |
| Spencer 2008 | 2 | 1 | 1 | 0 | 4 |
| Vogel 2006 | 2 | 1 | 1 | 0 | 4 |
| Vogel 2007 | 2 | 1 | 1 | 0 | 4 |
| Yeates 2020 | 2 | 1 | 0 | 0 | 3* |
| Zhu 2018 | 2 | 1 | 1 | 1 | 5 |
| *Excluded from data extraction for high risk of bias | | |  |  |  |
