# Supplementary material for: Is there a maternal blood biomarker that can predict spontaneous preterm birth prior to labour onset? A systematic review
Source: PLoS One. 2022 Apr 4;17(4):e0265853. doi: 10.1371/journal.pone.0265853 (PMC8979439; doi:10.1371/journal.pone.0265853)
Supplement: S3 File — (PDF) [file pone.0265853.s003.pdf]

Ref	Journal	Author	Year	objectives	participant characteristics	participant numbers	setting/context	study year range	Tissue	Method of measurement	Timepoint	outcome of interest	study design	country of origin	Biomarkers measured	significance	results/direction	heterogeneity
12	Journal of Obstetrics and Gynaecology	Abdel Malek et al.	2018	evaluate the value of measurement of serum ferritin levels in pregnant women to predict preterm labour	haemoglobin level >10.5gm/dl<br>mean age 29<br>mean parity 2.6<br>mean BMI 28<br>mean GA at delivery PTB 33.45 (1.58 SD)<br>mean GA at delivery TB 38.86 (1SD)	n=23 PPROM<br>n=17 sPTB<br>n=196 TB	outpatient clinic and inpatient ward of Obstetrics and Gynecology department at Kasr Al-Aini Hospital, El-Zahraa Teaching Hospital and El-Sahel Teaching hospital	January 2016-June 2016	serum	ELISA	30 weeks	PTB <37 weeks (with or without PPROM)	cohort	Egypt	ferritin<br>haemoglobin	y<br>n	elevated serum ferritin associated with sPTB	no differences with respect to Hb and ferritin levels in primigravida group and multigravida group at term or preterm
13	Scientific Reports	Akoto et al.	2020	investigate the association between HIV infection in human pregnancy, preipheral innate lymphoid cell frequencies and adverse pregnancy outcomes, specifically preterm birth	black south african aged 18 and over with singleton spontaneous pregnancy<br>mean age 30<br>mean parity 2<br>history of adverse pregnancy outcome 58%<br>mean BMI 27<br>smoking during pregnancy 7%<br>alcohol during pregnancy 13%<br>education 12yrs	n=46 HIV+<br>n=45 HIV-	prospective pregnancy cohort study at Chris Hani Baragwanath Academic Hospital	November 2013-October 2015	peripheral blood mononuclear cells	flow cytometry	8-14, 23-28, 30-39 weeks	PTB <37, 32-36, 28-31, 16-28 weeks	cohort	South Africa	ILC1<br>ILC2<br>ILC3	n<br>y<br>y	ILC2 and ILC3 frequency significantly lower in sPTB in all women, same trend in ILC1 but nonsignificant	reduced ILC is associated with sPTB - not different in HIV- and HIV+ women<br>ILC levels lowest in extreme PTB
14	American Journal of Obstetrics and Gynecology	Alleman et al.	2013	create a predictive model for preterm birth from available clinical data and serum analytes	women with singleton pregnancies with first and second serum analytes measured<br>patient demographics not described	n=2499 TB<br>n=200 PTB (including n=153 sPTB)	women undergoing first trimester screening	not stated	serum	unclear - routine serum screening (quad screen) and lipid diagnostic panel	first and/or second trimester	PTB <37 weeks without PPROM	case-control	USA	PAPP-A<br>hCG<br>estriol<br>AFP<br>inhibin A<br>total cholesterol<br>LDL<br>HDL<br>triglycerides<br><br>AFP, inhibin A, total cholesterol T1, total cholesterol T2-T1	n<br>n<br>n<br>y<br>y<br>n<br>n<br>n<br>n<br><br>y	high total cholesterol, low change in cholesterol from T1 to T2, high AFP and high inhibin A association with sPTB	group with previous sPTB as a predictor had better prediction than nulliparous or parous women with no previous sPTB
15	Environment International	Ashrap et al.	2020	investigate the effects of matl(loid)s on adverse birth outcomes bith individually and as mixtures	women 18-40 years living in Northern Karst aquifer region, no major medical or obstetrical complications including diabetes, no use of oral contraceptives of in vitro fertilization 3mo before pregnancy	n=812	PROTECT cohort for exploring contamination sites, high rate of PTB	2010	plasma	mass spec	16-20 weeks<br>24-28 weeks	sPTB <37 weeks	cohort	Puerto Rico	Co<br>Zn<br>Pb<br>Cs<br>Cu<br>Mn<br>Ni<br>As<br>Cd<br>Hg	y<br>y<br>y<br>n<br>n<br>n<br>n<br>n<br>n<br>n	high levels of metal(loid)s was associated with higher odds of sPTB	fetal sex specific interactions were not observed
16	Scientific Reports	Aung et al.	2019	characterize the associations and predictive capacity of an extensive panel of eicosanoids, immune biomarkers, oxidative stress markers, and growth factors towards preterm birth and its subtypes	controls had lower BMI, higher education level and were predominantly white compared to the cases but not significant. Majority were privately insured	n=31 sPTB<br>n=115 TB	LIFECODES prospective birth cohort at the Brigham and Women's Hospital in Boston	2006-2008	plasma	ELISA, bead based panel, immunoassays, mass spec	23.1-28.9 weeks	sPTB <37 weeks (with or without PPROM)	case-control	USA	LA, AA, EPA, DHA, 12-HETE, 15-HETE, 12-oxoETE, 5-oxoETE, LTC4-ME, LTE4, LTD4, 5-HETE, RVD1, RVD2, 11,12-DHET, 9(10)-Epo-ME, 12(13)-EpoME, 5(6)-EET, 11(12)-EET, 8(9)-EET, 8,9-DHET, 9,10-DiHOME, 12,13-DiHOME,<br>9-oxoODE, PGB2, 13,14DHK-PGF2a, PGJ2, 15DO12,14-PGJ2, PGE2, 13,14-DHK-PGD2<br><br>CRP,TNfa, IL-10, IL-6, IL-1B, DY, CY, NY, sFLT-1, PGF, 8-OHdG, 8-IP, L1B4, 15-oxoETE, 13-oxoETE, 13S-HODE, 9S-HODE, 5,6-DHET, 18-HETE, 17-HETE, 16-HETE, 11-HETE, 20-HETE, CAA, 14(15)-EET, PGE1, BCPGE2, BCPGE2, PGE3, PGD3, PGA2, PGD2, TXB2<br><br>5-HETE, LTD4, LTC4-ME, 13-oxoODE, 5-oxoETE, 8-HETE, LTB4	y<br><br>n<br><br>y	lipid biomarkers performed best at identifying PTB, specifically lipoxygenase and cytochrome p450 markers	na
17	Journal of Maternal-Fetal and Neonatal Medicine	Bakalis et al.	2012	examine the potential value of maternal serum level of CRP in the first trimester of pregnancy in the prediction of spontaneous early preterm delivery	mean age 32, median weight 63kg, median height 164cm, mostly caucasian, primarily spontaneous conception, primarily nulliparous<br>cases were more likely to have a previous preterm delivery	n=30 sPTB<br>n=90 TB	prospective study for adverse outcomes in women attending first routine hospital visit in pregnancy	not stated	serum	immunoassay	11-13 weeks	sPTB <34 weeks	case-control	UK	CRP	n	CRP was not different in terms compared to preterms and not predictive	na
18	Journal of Perinatology	Bandoli et al.	2018	test whether second trimester serum cortisol is higher in spotnaneous preterm births compared to provider initiated (mediaclly indicated) preterm births	primarily hispanic or non-hispanic white, mean maternal weight 147lb, mostly 18-34 years	n=496 sPTB<br>n=495 TB	nested within Genetic Disease Screening Program for prenatal screening for aneuploidies	2009-2010	serum	mass spec	16.5 weeks	sPTB <32 weeks, 32-36 weeks	case-control	USA	cortisol	n	mean cortisol levels for each category of gestational length did not differ	among non-hispanic white mothers, cortisol was significantly higher among women who gave birth <32 weeks compared to >38 weeks, but not other racial groups
19	Fetal Diagnosis and Therapy	Beta et al.	2011	to examine the potential value of maternal serum level of AFP in the first trimester of pregnancy in the prediction of spontaneous early preterm delivery	median age 31, median weight 64kg, median height 165 cm, caucasian and african, non smoker, spontaneous conception, preterm group more likely to have a preterm delivery than term group	n=33 sPTB<br>n=99 TB	prospective study for adverse outcomes in women attending first routine hospital visit in pregnancy	not stated	serum	chemiluminescence	11-13 weeks	sPTB <34 weeks	case-control	UK	AFP and maternal characteristics	y	AFP is increased in preterm delivery and improves prediction based on maternal and obstetric characteristics alone	AUROC was higher in parous women and lower in nulliparous women compared to all women
20	Prenatal Diagnosis	Beta et al.	2011	develop a model for prediction of spontaneous delivery before 34 weeks based on maternal factors, placental perfusion and function at 11-13 weeks' gestation	median age 32, median weight 66kg, median heigh 163cm, primarily caucasian, spontaneous conception, non smoker, preterms were more likely to have a previous PTB or miscarriage	n=353 sPTB<br>n=33017 TB	prospective study for adverse outcomes in women attending first routine hospital visit in pregnancy	not stated	serum	ELISA and immunoassay	11-13 weeks	sPTB <34 weeks (with or without PPROM)	case-control	UK	PAPP-A<br>B-hCG<br>P1GF<br>PP13<br>ADAM12<br>inhibin A<br>activin A	y<br>n<br>n<br>n<br>n<br>n<br>n	PAPP-A did not improve prediction model compared to maternal characteristics alone	PAPP-A did not improve model in nulliparous group
21	Journal of Maternal-Fetal and Neonatal Medicine	Beta et al.	2012	examine the potential value of maternal serum level of ferritin in the first trimster of pregnancy in the prediction of spontaneous early preterm delivery	median age 33, median weight 63kg, median heigh 164cm, majority caucasian, preterms more likely to have previous PTB	n=30 sPTB<br>n=90 TB	prospective study for adverse outcomes in women attending first routine hospital visit in pregnancy	not stated	serum	immunoassay chemiluminescence	11-13 weeks	sPTB <34 weeks	case-control	UK	ferritin	n	ferritin not significantly different in sPTB compared to term	na
22	Clinical Mass Spectrometry	Bradford et al.	2017	deveop a second trimester test predictive of sPTB	women with singleton pregnancies aged 18-60 years, receiving prenatal care, no known or suspected fetal anomaly	n=413 and serum pools from pregnant and non-pregnant donor	Proteomic assessment of Preterm Risk (PPAR) study	2014-2015	serum	tandem mass spectrometry	17-28 weeks	sPTB <37 weeks	cohort	USA	IBP4<br>SHBG	y<br>y	IBP4/SHBG predictive of sPTB	na
23	Reproductive Sciences	Bullen et al.	2013	examine the association between CRP and PTD pathways sPTD or miPTD. Plus looked at chorioamnionitis and effect of BMI	singleton pregnancy, no known chromosomal abnormality or birth defect, MSAFP screening, age >= 15 years, no prepregnancy diabetes mellitus, proficient in English, mostly white or african american, more than half with prepregnancy BMI overweight/obese, 42% nulliparous, 43% never been Medicaid insured	n=1310	Pregnancy Outcomes and Community Health study for study of adverse pregnancy outcomes	sept 98 - june 2004	plasma	ELISA	16-27 weeks	sPTB <37 weeks (with or without PPROM)	cohort	USA	CRP	y	CRP significantly higher in sPTB compared to term (p=0.02)	CRP was associated with sPTD, due in part to Chorioamnionitis. Also higher in women with BMI>25 with or without CA
24	American Journal of Obstetrics and Gynecology	Cantonwine et al.	2016	examine potential differences in plasma microparticle proteins at 10-12 weeks in htose that delivered PT<34 weeks compared to T	mostly white or hispanic, mean age 32, mean BMI 28, controls had higher maternal education levels, preterms were more likely to be primaparous and have a history of preterm birth	n=25 sPTB<34 weeks<br>n=50 term	prospective	2009-2014	plasma microparticles	mass spec	10-12 weeks	sPTB<34 weeks	case-control	USA	AACT, KLKB1, APOM, ITIH4, IC1, KNG1, TRY3, C09, F13B, APOL1, LCAT, PGRP2, THBG, FBLN1, ITIH2, CD5L, CBPN, VTDB, AMBP, C08A, ITIH1, TTHY, F13A, APOA1, HPT<br><br>the above list is the top 25 proteins associated with sPTB. 62 of 132 proteins evaluated had robust power of detecting sPTB, but only 25 were shown	y	62/132 proteins can detect sPTB using ROC analysis	na
25	American Journal of Epidemiology	Catov et al.	2014	investigate whether NEFAs mark elevated risk for sPTB by assessing concentrations of NEFAs before 21 weeks of gestation in women with and without sPTB	mostly nulliparous, median age 23, median prepregnancy BMI 24.7, %34 black	n=115 sPTB n=222 TB	prospective from clinics and private practices in Pittsburgh Pennsylvania	1997-2001	serum	lipid assay, immuno assay for CRP	4-20 weeks	sPTB 34-36 weeks<br>sPTB <34 weeks	case-control	USA	total cholesterol<br>triglycerides<br>HDL<br>LDL<br>CRP<br>uric acid<br>NEFA<br><br>NEFA, HDL	n<br>n<br>n<br>n<br>n<br>n<br>y<br><br>y	Higher levels of NEFAs with HDL associated with sPTB	family history of gestational hypertension

26	Metabolites	Considine et al.	2019	investigate the potential of maternal serum to provide metabolomic biomarker candidates for the prediction of spontaneous preterm birth in asymptomatic pregnant women at 15 and/or 20 weeks gestation	mean age 30, mean BMI 24-25, all nulliparous	n=50 sPTB n=55 TB	women who participated in the screening for obstetric and pregnancy endpoints (SCOPE) study in Cork	2011	serum	liquid chromatography mass spectrometry	15 and 20 weeks	sPTB <37 weeks	case-control	Ireland	bile acid metabolite<br>prostaglandin metabolite<br>vitamin D metabolite<br>fatty acid metabolite	y<br>y<br>y<br>y	4 features produce a biomarker panel that predicts sPTB	Suggest BMI linked to some biomarkers
27	Acta Obstetricia et Gynecologica Scandinavica	Curry et al.	2007	evaluate the relationship between elevated mid-pregnancy levels of maternal plasma IL2, IL6, TNFa, IFNy, and GM-CSF and early, noderate and late sPTB. Additionally, sought to determine whether associations vary by pre-pregnancy BMI, maternal age, gravidity, and prior PTD	most had BMI within 18.5-24.9, mostly <35 years old, 40-50% alcohol use during pregnancy, preterms more likely to have a prior preterm delivery, spontaneous abortion, or induced abortion, and to be multigravid and nulliparous, early or moderate preterms were more likely to be >= 35 years old than controls, early preterms were more likely to be smokers than controls	n=61 early sPTB, n=278 moderate sPTB, n=334 late sPTB, n=1125 TB	case control nested in Danish National Birth Cohort	1997-2002	plasma	multiplex flow cytometric assay liminex multianalyte profiling tehcnology	>17 weeks	sPTB <24-29 wks, 30-33 wks, 34-36 wks, and >37 wks	case-control	Denmark	IL2<br>IL6<br>TNFa<br>IFNy<br>GM-CSF	n<br>y<br>n<br>y<br>n	IL6 and IFN weak association with late sPTB, but not the early or moderate PTB	early sPTD smokers,
28	Acta Obstetricia et Gynecologica Scandinavica	Curry et al.	2009	examine associations with 1st trimester cytokines and sPTB	most had BMI within 18.5-24.9, mostly <35 years old, 40-44% alcohol use during pregnancy, preterms were more likely than terms to have had a prior preterm delivery, a prior induced abortion, be multigravid and nulliparous and report serious medical disease, early or moderate preterms were more likely to have had a prior spontaneous abortion and be >= 35 years old, moderate preterms were more likely than terms to have a BMI <25	n=107 early sPTB, n=353 moderate sPTB, n=422 late sPTB, n=1372 TB	case control nested in Danish National Birth Cohort	1997-2002	plasma	multiplex flow cytometric assay luminex multianalyte profiling tehcnology	8 weeks	sPTB <24-29, 30-33, 34-36, and >37 weeks	case-control	Denmark	IL2<br>IL6<br>TNFa<br>IFNy<br>GM-CSF	y<br>y<br>y<br>n<br>y	TNF & GM-CSF associated with late sPTB (34-36 wks).	underweight and obese
29	Journal of Maternal-Fetal and Neonatal Medicine	Dhaifalah et al.	2014	determine any association between azurocidin in 1st trimester with subsequent sPTB, PPROM and iPTB	mean age 30, primarily non smokers, spontaneous preterms were more likely than terms to be primiparous, but not significant	n=13 sPTB, n=17 PPROM, n=22 TB	cross sectional cohort study	2011	serum	ELISA	11-13 weeks	sPTB <37 weeks	case-control	Czech Republic	azurocidin	y	low azurocidin associated with PPROM compared to all - sPTB, iPTB and term delivery	PPROM
30	Fetal Diagnosis and Therapy	El-Achi et al.	2020	identify risk factors for PPROM. Develop a 1st trimester prediction model	primarily caucasian and asian, no preexisting diabetes, non smokers, mean age 32.7, mean BMI 24.6, 58% nulliparous, PPROMs were more likely to have preexisiting diabetes mellitus, be older and have higher BMI	n=144 PPROM	retrospective analysis of 1st trimester screening for aneuploidy and preeclampsia	April 2010-October 2016	serum	ELISA?	11-13+6 weeks	PPROM	cohort	Australia	PAPP-A<br>B-hCG<br><br>PAPP-A (in model)	n<br>n<br><br>y	low PAPP-A lower risk of PPROM compared to high PAPP-A	nulliparity, preexisting DM, type 2DM, higher maternal age and BMI increased risk of PPROM
31	American Journal of Obstetrics and Gynecology	Esplin et al.	2011	to identify serum markers of sPTB in asypmtomatic women before labor	mean age 24, approx. 30% nulliparous, approx 70% african american	n=40 sPTB, n=40 TB collected at 24 wks. n=40 sPTB, n=40 TB collected at 28 wks	nested case control, part of Preterm Prediction Study	1992 - 1994	serum	mass spec	n=80 sampled at 24 and additional n=80 at 28 weeks	sPTB <35 wks	case-control	USA	PGF<br>TAT<br>CRH<br>defensin<br>ferritin<br>lactoferrin<br>TNFR1<br>ITIH4	n<br>y<br>y<br>y<br>y<br>y<br>y<br>y	three proteomic peptides arising from ITIH4 were reduce in women at 24 and 28 weeks having subsequent sPTB combared to term	na
32	American Journal of Perinatology	Ezrin et al.	2015	determine if a proteomic biosignature of circulating microparticles from 2md trimester serum can identify sPTB	all primigravida or second pregnancy, no history of PTB, mean age 28 for primigravida, 32 for second pregnancy, mostly non smokers, no diabetes	n=24 sPTB, n=24 TB	required 1yr post screening?	not stated	serum	liquid chromatrography mass spec	15-17 weeks	sPTB<34 wks	case-control	USA	A2M, C1R, C3, CFH, SERPINA1, A1BG, ALB, APOL1, C4, IGHM2, APOD, AZGP1, HPX, IGHA1, IGHM1, IGGk, SERPINC1, TF<br><br>18 'top' biomarkers of 99 proteins that were identified as differentially expressed in sPTB and term	y	99 proteins were significantly different in term compared to sPTB, 18 biomarkers were evaluated	na
33	American Journal of Reproductive Immunology	Ferguson et al.	2014	compare cytokine profiles over 4 visits in pregnancy with normal and preterm pregnancies	predominantly white (59%), education level (68% junior college, some college or college graduate) and non smokers (92%), majority publicly insured, mostly BMI <25	n=350 TB PTB subgroups n=56 sPTB, n=35PET or IUGR	nested case control,	2006-2008	plasma	ELISA and bead based assay	4.71-16.1 and 14.9-21.9 and 22.9-29.3 and 33.1-38.3 weeks	PTB<37 weeks	case-control	USA	IL1B<br>IL6<br>IL10<br>TNFa<br>CRP	n<br>y<br>n<br>n<br>n	IL6 was associated with increased odds of sPTB, associations strongest later in pregnancy	Association of CRP higher in African American compared to Caucasion, high school eductaion compared to college degree, public compared to private health insurance, and BMI>25 kg/m2. Increased levels of IL6, IL10 and TNFa associated with overall PTB. IL6 stronger predictor of sPTB, IL10 stronger predictor in placental PTB
34	American Journal of Obstetrics and Gynecology	Goldenberg et al.	2000	determine if G-CSF measured in asymptomatic women at 24 and 28 weeks is associated with sPTB at <32 weeks	predominantly 16-35 years old, 72.7% african american, 33.5% nulliparous, preterms were more likely to have BMI <19.8 and to have had pulmonary disease, a previous spontaneous preterm birth, vaginal bleeding, or a urinary track infection	n=194 sPTB, n=194 TB collected at 24 weeks. Of these 132 had additional sample at 28 weeks	nested case control as part of Preterm Prediction Study	1992 - 1994	plasma	ELISA	24 and 28 week collection	sPTB<32 weeks	case-control	USA	G-CSF	y	elevated G-CSF is associated with early sPTB	elevated G-CSF at 24 and 28 weeks associated with sPTB at <32 weeks but not later sPTB
35	American Journal of Obstetrics and Gynecology	Goldenberg et al.	2001	in asymptomatic women identify biomarkers associated with sPTB	the cohort that cases and controls were selected from was 63% black, 58% multiparous, and mean age was 23.7, within cases and controls: preterms were more likely to have had a previous spontaneous PTB	n=50 sPTB <32 weeks, n=127 sPTB <35 weeks matched term controls	nested case control as part of Preterm Prediction Study	1992 - 1994	serum	unreported	24 and 28 weeks	sPTB<32 and sPTB<35 weeks	case-control	USA	CRH<br>AFP<br>alkaline phosphatase<br>B2M<br>ferritin<br>ICAM-1<br>IL6<br>CRP<br>cortisol<br>lactoferrin<br>defensins<br>relaxin<br>IL10<br>G-CSF<br>activan<br><br>AFP, alkaline phosphatase, G-CSF, defensins	n<br>y<br>y<br>n<br>n<br>y<br>n<br>n<br>n<br>n<br>y<br>n<br>n<br>n<br>n<br><br>y	combining 5 markers (cervical fFN, cervical length, AFP, alkaline phosphatase, G-CSF) was predictive of sPTB	With sPTB<32wks, significant association with AFP, alkaline phosphatase, ICAM-1, and defensins, but with sPTB<35 weeks only AFP and alkaline phosphatase.
36	Journal of Obstetrics and Gynaecology Research	Gupta et al.	2015	determine if low PAPP-A associated with adverse pregnancy outcomes	mean age 25, mean weight 53 kg, approx half nulliparous, all non smokers,	n=130 with low PAPP-A, n=200 with normal range PAPP-A as controls	North Indian cohort	Aug 2010 to Dec 2011	serum	fluorometry	9-13 weeks	PTB <37 weeks, FGR, PET, oligohydramnios, low birthweight	case-control	India	PAPP-A	y	incidence of PTB was higher in women with low PAPP-A	low PAPP-A asscoiated with FGR, oligohydramnios and low birthweight. This was less significant for preterm delivery
37	American Journal of Obstetrics and Gynecology	Hackney et al.	2010	determine association of thrombin-antithrombin (TAT) in asymptomatic women at 24 and 28 weeks and sPTB	mean age 23, 72.1% black, preterms were more likely to have lower BMI, prior vaginal bleeding and a prior sPTB	n=169 matched sPTB/TB pairs at 24 weeks, n=138 at 28 weeks	nested case control as part of Preterm Prediction Study	1992 - 1994	serum	immunoassay	23-24 and 27-28 weeks	sPTB <37 weeks	case-control	USA	TAT	y	TAT was lower in sPTB compared to term at 28 weeks, but not at 24 weeks	TAT levels measured at 28wk but not at 24 weeks was associated with sPTB at <37 weeks,
38	PLoS ONE	Heng et al.	2016	examine the expression profile in assymptomatic women, collected at 17-23weeks and 28-33 weeks and determine if predictive of sPTB	mean age 31, primarily caucasion and non smokers, preterms were more likely than terms to have a history of previous PTB, history of abortion, UTI during pregnancy, anaemia during pregnancy and have group B streptococcus in vaginal tract >36 weeks ga	n=51 sPTB, n=114 TB	longitudinal cohort	May 2008 - Dec 2010	RNA isolated from whole blood	affymetrix multiplex microarray of RNA	17-33 weeks	sPTB <37 weeks	case-control	Canada	top of three models contained the following gene features:<br><br>*LOC100128908, MIR3691, LOC101927441, CST13P, ACAP2, ZNF324, SH3PXD2B, TBX21*	y	model using the difference in gene expression at two timepoints can predict sPTB with 65% sensitivity and 88% specificity	antepartum haemorrhage, urinary tract infection during pregnancy, anaemia

39	Cytokine	Huang et al.	2019	to identify if a combination of maternal serum cytokines is predictive of sPTB<37 weeks	mean age 28, majority Han Chinese, non smokers, multigravid, high school or less education, distribution of prepregnancy BMI was different between preterms and controls	n=129 sPTB, n=258 matched control TB	nested case control	2015 - 2016	serum	multiplex cytokine array (Bioplex 200 system)	range from 13 weeks, 14-27 weeks or ≥28 weeks	sPTB at <34 weeks and <37 weeks	case-control	China	IL10<br>MCP-3<br>TRAIL<br>TNFa<br>IL1B, IL1Ra, IL2, IL2Ra, IL4, IL5, IL6, IL7, IL8, IL9, IL12, IL13, IL15, IL16, IL17, IL18, Eotaxin, FGF-bas-c, G-CSF, GM-CSF, IFNy, IP10, MCP-1, MIP-1a, PDGF-B, MIP-1B, RANTES, VEGF, MIF, TNF-B HGF	y<br>y<br>y<br>y<br>n	higher levels of IL-10, MCP-3, TRAIL and TNF-a associated with sPTB, after adjusting for age, BMI, race, medication,income and education	predictive performance was improved by high BMI
40	Biomedical Journal	Huang et al.	2020	Determine if inhibin is predictive of PTB	mean age 30, mean weight 58 (kg?)	n=687 at term >37 weeks and n=83 PTB <37 weeks	retrospective cohort study	2011-2015	blood centrifuged not stated if serum or plasma	ELISA kits	15-20 weeks	sPTB and or PPROM <37 weeks	cohort		AFP<br>hCG<br>uE3<br>inhibin A	n<br>n<br>n<br>y	higher levels of inhibin A in the PTB group compared to term	na
41	Acta Obstetricia et Gynecologica Scandinavica	Hvilsom et al.	2002	Determine if CRP in second trimester is associated with preterm delivery	mean weight 63 kg, mean height 167 cm, mean BMI 22, preterms were more likely to be younger and to have had a previous preterm delivery	n=84 sPTB, n=400 controls	multipurpose cohort study	Nov 1992 - Feb 1994	serum	ELISA	16 weeks	sPTB (gestation not defined)	case-control	Denmark	CRP	y	median CRP levels higher in women delivering PT compared to term	na
42	Journal of Maternal-Fetal and Neonatal Medicine	Inan et al.	2018	To compare PROK1, PAPP-A andPROK1/PAPP-A ratios in the 1st trimester and ability to predict adverse pregnancy outcomes - including sPTB	all caucasian, age range 18-42, median age 31, all non smokers, no alcohol intake	n=16 sPTB, n=102 TB	1st trimester screening	March 2015 - July 2016	serum	ELISA	11-13 weeks	sPTB<37 weeks	cohort	Turkey	PROK-1 (aka EG-VEGF)<br>PAPP-A<br>PROK-1/PAPP-A<br>PROK-1, PROK-1/PAPP-A	y<br>n<br>y<br>y	lower PROK1 levels and PROK1/PAPP-A ratios in first trimester are associated with sPTB	na
43	Prenatal Diagnosis	Jelliffe-Pawlowski et al.	2010	examine relationship between 2nd trimester maternal serum biomarkers and PTB	majority hispanic or white between 18 and 34 years old, 35.6% nulliparous, 35% receiving state funded health care services, all non smokers, no history of diabetes, body weights at prenatal screening between 10th and 90th percentile for gestational age/race-ethnicity grouping	n=102861 in cohort. n=1116 early PTB, n= 6933 late PTB	California Expanded AFP screening program administered by the genetic disease screening program	2006	serum	Auto DELFIA	TM2	sPTB 20-37, 20-32, 33-36 weeks	cohort	USA	AFP<br>hCG<br>uE3	y<br>y<br>y	elevated levels of AFP, hCG and uE3 asscoiated with PTB. Risk increased if more than one biomarker elevated	na
44	American Journal of Obstetrics and Gynecology	Jelliffe-Pawlowski et al.	2013	investigate whether preterm birth is associated with single an dmultiple biomarker abnormalities	mostly hispanic and between 18 and 34 years old, preterms were more likely to be of black race/ethnicity	n=345 early sPTB, n=1725 TB	California prenatal screening program and the Californa perinatal quality care collaborative	2009-2010	serum	Auto DELFIA	10-15 and 15-20 weeks	sPTB <37 weeks	case-control	USA	PAPP-A<br>hCG<br>AFP<br>uE3<br>inhibin A	y<br>n<br>y<br>n<br>y	low PAPP-A in TM1, high AFP in TM2, high INH in TM2 were associated with higher risk of sPTB	models excluding PROM performed similarly to models including PROM
45	International Journal of Obstetrics and Gynaecology	Jelliffe-Pawlowski et al.	2015	examine the relatinship between amternal charactersitics, serum biomarkers and preterm birth	primarily hispanic or non hispanic white and between 18 and 34 years old, preterms were more likely to have had a previous PTB	n=125202	California Officie of Statewide Health Planning and Development database first trimester serum screening	2009-2010	serum	Auto DELFIA	11-20 weeks	sPTB 20-31 weeks, or 32-36 weeks	cohort	USA	PAPP-A<br>hCG<br>AFP<br>uE3<br>inhibin A	y<br>n<br>y<br>y<br>y	ten maternal characteristics, low PAPP-A in TM1, high AFP in TM2, and high INH in TM2 were associatiated with 51.6-86.2% increased of PTB	associations of biomarkers with PTB tended to be higher for early PTB (<32 weeks)
46	Journal of Perinatology	Jelliffe-Pawlowski et al.	2018	To develop a comprehensive test for PTB across subtypes including preeclampsia could be devleoped using mid-pregnancy growth and immune related factors along with maternal demographics and obstetric factors	primarily hispanic or white and between the ages of 18 and 34, born in the US, >= 12 years of education, approx 40% low income, approx half nulliparous, 20% obese, preterms were more likely to be low income than terms	n=200 sPTB, n=200 TB	routine prenatal screening for aneploiding by Califorina genetic disease screening program	2009-2010	serum	Luminex 200	15-20 weeks	PTB <37 weeks (sPTB or with preeclampsia)	case-control	USA	PAI1, resistin, GP130, ENA-78, sFASL, FGF-basic, G-CSF, IL1R2, IL4, IL4R, IL5, IL13, IL17, IL17F, IFNB, M-CSF, NGF, PDGFBB, RAGE, SCF, VEGFR3, eotaxin, MIG, MIP-1B, RANTES<br>IL1A, IL1RA, IL1B, IL2, IL2RA, IL6, IL6R, IL7, IL10, IL12p40, IL12p70, IL15, IFNA, IFNy, MCP-1, MIP-1A, MCP-3, GRO-A, IL8, IP-10, TNFa, TNFR2, CD30, CD40L, TNFB, TRAIL, TGFA, TGFB, SCF, LIF, NGF, VEGF VEGFR1, VEGFR2, HGF, GM-CSF, G-CSF, sVCAM-1, sICAM-1, leptin, RAGE	y<br>n	25 serum biomarkers along with maternal age and pverty status identify 80% of women with PTB +/- preeclampsia	detection was generally better for PTB <32 weeks than it was for PTB 32-36 weeks
47	Journal of Maternal-Fetal and Neonatal Medicine	Kansu-Celik et al.	2019	evaluate the value of maternal serum advanced glycation end products (AGEs) level at 11013 weeks gestation for the prediction of preterm labour and/or preterm premature rupture of membranes (PPROM)	mean age 28.7, median gravidity 2, median parity 1, mean BMI 27.86, all non smokers, no history of preterm delivery or PPROM, no chronic diseases diagnosed before conception, no gestational diabetes or preeclampsia	n=21 spTB, n=25 TB	routine antenatal follow-up at university affiliated hospital	2016	serum	ELISA	11-13 weeks	sPTB <37 weeks with or without PPROM	case-control	Turkey	AGE	y	first trimester AGEs were significnatly higher in PTL/PPROM compared to control sensitivity 91.7 and specificity 73.8	no difference in AGE levels between nulliparous and parous women
48	British Journal of Nutrition	Khambalia et al.	2015	examine the association between Fe biomarkes and the risk of total, early and moderate to late sPTB	primarily 25-34 years old, born in Australia, 6-7% smoking in pregnancy, approx half nulliparous, 2% gestational diabetes, 4% hypertensive disorders in pregnancy, preterms were more likely to be heavier and have GDM	n=2254 sPTB	women attending first trimester Down's syndrome sreeening	2007	serum	ELISA	11-13 weeks	sPTB <37 weeks with or without PPROM, <34 weeks and 34-36 [illegible]	cohort	Britain	ferritin<br>sTfr<br>CRP	y<br>n<br>y	increased odds of sPTB for women with elevated ferriting levels	association between serum ferritin >90th percentile and sPTB was stronger for early PTB (<34 weeks) than for moderate to late PTB
49	Prenatal Diagnosis	Kirkegaard et al.	2010	evaluate early featl growth and the biomarkers PAPP-A, BhCg in relation to preterm delivery	primarily <35 years old, BMI<30, 53% multiparous, 4% IVF pregnancy	n=9450	prenatal screening program at Aarhus University Hospital	2005-2007	serum	secondary data (national database)	8-13 weeks	sPTB <37 weeks	cohort	Denmark	PAPP-A<br>B-hCG	y<br>y	low PAPP-A and low free B-hCG were significantly associated with PTD, even stronger with combined with slow early fetal growth. PAPP-A and B-hCG were not assocaited with PTD with fast early fetal growth	low PAPP-A values were more strongly associated with PTB in pregnancies carryign a female fetus compared to a male fetus
50	Prenatal Diagnosis	Kirkegaard et al.	2011	evaluate whether PAPPA and free B-hCG before 10 weeks of gestation affects the association between these biomarkers and adverse pregnancy outcomes	primarily <35 years old, maternal height between 160-170 cm, 53% multiparous	n=9450	prenatal screening program at Aarhus University Hospital	2005-2007	serum	secondary data (national database)	<10 weeks	sPTB <37 weeks	cohort	Denmark	PAPP-A<br>B-hCG	y<br>y	b-hCG had a stronger associated with PTD with early sampling, but PAPP-A did not	na
51	Australian and New Zealand Journal of Obstetrics and Gynaecology	Kwik et al.	2003	to investigate whether low PAPP-A in TM1 are associated with subsequent intrauterine fetal growth restriction, stillbirth and PTD	singleton pregnancies who had serum screening carried out at the time of their nuchal translucency ultrasound, mean age 32.7, no other demographic information	n=827	patients who had serum screening carried out at the time of their nuchal transclucency ultrasound	not stated	serum	ELISA	11-13 weeks	sPTB <37 weeks	cohort	Australia	PAPP-A	n	low PAPP-A levels was not associated with PTB	na
52	British Journal of Obstetrics and Gynaecology	Leung et al.	1999	test whether maternal CRH levels are elevated in the mid trimster for those women who subsequently had sPTB and to assess the clinical utility of the measurement in the prediction of PTD	mean age 30.8, 47% nulliparous, majority ethnically Chinese	n=1047	department of obstetrics and gynaecology, Prince of Wales hospital, Hong Kong	1996-1997	plasma	radioimmunoassay	15-20 weeks	sPTB <34 weeks	cohort	Hong Kong	CRH	y	CRH was significantly higher in sPTB compared to term, prediction produced a sensitivity of 72% specificty 78	midtrimester CRH was significantly different between early PTB (<34 weeks) and term birth but not between late TPB and term birth

53	American Journal of Obstetrics and Gynecology	Lynch et al.	2016	to perform a large scale biomarker discovery and to determine which protein pathways are most strongly associated with PTB	mean age 34, primarily non hispanic white, 46% nulliparous, preterms were more likely than terms to be a race other than non hispanic white	n=22 sPTB, n=88 TB	Denver Complement Study	not stated	plasma	SOMAscan proteomic assay	10-15 weeks	sPTB 20-37 weeks with or without PPROM	case-control	USA	coagulation factor IX, coagulation factor IX ab, factor B, PECAM-1, Factor H, SAP, VEGF sR2, CATZ, growth hormone receptor, ficolin-3, ATS15, Pcadherin, MP2K2, cathepsin A, leptin, C5a, PIGR, calcineurin Ba, cGMP-stimulated PDE, TLR2, sogg-1, RET, JAG1, MMP-2, Notch-3, angiopoietin-2, MAPKAPK3, PAPP-A, FN1.3, IGFBP-2, fibronectin, MP2K4, hemopexin<br><br>above are the proteins significantly related to PTB from univariate analysis (of 1129 proteins measured)	y	complement factors B and H and coagulation factor IX were the highest ranking proteins distinguishin PTB from term	na
54	Journal of Clinical Laboratory Analysis	Ma et al.	2020	study whether complete blood count (CBC) parameters at 20-30 weeks of pregnancy can predict asymptomatic PTB	mean age 30, mean prepregnancy BMI 20, mean gravidity 1.7, mean parity 0.3	n=105 sPTB, n=210 TB	Fujian Provincial Maternity and Children's Hospital	2017	whole blood	Sysmex-SN3000 blood cell counter	20-30 weeks	sPTB 28-36 weeks	case-control	China	whole blood count<br>neutrophil count<br>lympcyte count<br>monocyte count<br>hemoglobin<br>hematocrit<br>red cell distribution width<br>platelet count<br>mean platelet volume<br>plateletcrit<br>platelet distribution width<br>neutrophil to lymphocyte ratio<br>lymphocyte to monocyte ratio<br>platelet to lymphocyte ratio	n<br>n<br>y<br>n<br>y<br>y<br>n<br>n<br>y<br>n<br>y<br>y<br>y<br>y	neutrophil to lymphocyte ratio, hemoglobin, platelet distribution width in combination predicts PTB with sensitivity and specificity of 88% and 41%	na
55	Epigenomics	Manuck et al.	2021	measure whether changes in RNA in mother's blood in early pregnancy are predictive of early delivery	women at high risk of spontaneous preterm delivery, 34.6% non hispanic black race, mean age 31.8, 64.9% married, 34.3 low socioeconomic status, 11.2 % smoking in pregnancy, median prepregnancy BMI 29.3, 32.4% nulliparous, 91.3% prior PTB, preterm births <34 weeks were more likely to be nulliparous than term births, preterms were LESS likely to have had a prior PTB than terms	n=68 sPTB, n=68 TB	UNC PTB Biobank cohort study	2015-2017	whole blood	212-gene custom nanostring mRNA panel and custom 108 gene Nanostring miRNA panel	<24 weeks	sPTB <37 and <34 weeks	case-control	USA	212 mRNAs and 108 miRNAs in the NO pathway were evaluated<br><br>*TLR2, DUSP1, RUNX3, PPP3CA, B2M, TLR4, IL10RA, PREX1, CYC1, CHL1, CXCR3, IL10RB, DDAH2, RNF7, HSP90AB1, NCF1, MSRA, SDHA, GLA, NCOA2*	y	fourteen genes were differentially expressed in women delivering <37 weeks, 13/14 were also differentially expressed in those delivering <34 weeks compared to term. Gene expression improved clinical prediction models	CYC1 was associated with PTB <37 weeks but not PTB >34 weeks; more perturbations were seen in miRNA expression for PTB <34 weeks than in PTB <37 weeks
56	PLoS ONE	McDonald et al.	2015	examine whether levels of inflammatory and angiogenic mediators, measured early in pregnancy, were predictive of sPTB	primigravid singletons taking daily doses of iron and folic acid, and presumptive anti-malarial therapy with sulfadoxine-pyrimethamine tablets at 20 and 30 weeks gestation, all HIV negative, mean age 21, majority had 5-7 years of education, preterms were more likely to be younger, be unmarried, have fewer years of education, lower BMI and skinfold thickness, and lower socioeconomic status	n=432 training, n=646 test	multivitamin supplementation cohort for HIV negative women	2012-2013	plasma	ELISA	12-27 weeks	sPTB <37 and <34 weeks	cohort	Canada (Tanzanian Cohort)	Ang-1<br>Ang-2<br>AngptL3<br>VEGF<br>sFLT-1<br>sTNFR2<br>PGF<br>MIP-1B<br>MCP-1<br>Leptin<br>IL1B<br>IL18BP<br>sICAM-1<br>Factor D<br>sEndoglin<br>CRP<br>CHI3L1<br>C5a	n<br>y<br>y<br>n<br>y<br>y<br>y<br>n<br>n<br>y<br>n<br>y<br>y<br>n<br>y<br>n<br>y<br>y	IL-18BP, sICAM-1, dEndoglin and CHI13L1 were elevated and leptin was lower in those that experience sPTB. High leptin and Ang2 had reduced risk of sPTB	risk of early to moderate PTB increased with increasing CHI3L1, sICAM-1, and IL-18BP wherease risk of late PTB increased with increasing sTNFR2, CHI3L1, C5a, sICAM-1, and IL-18BP
57	American Journal of Obstetrics and Gynecology	McElrath et al.	2019	validate the circulating microparticle protein multiplex concept using a larger sample set to predict sPTB	mean age 31, 22% african american, mean BMI 28, majority privately insured, 10% smoking during pregnancy, mean parity 1, 49% male fetal sex	n=87sPTB, n=174 TB	Brigham and Women's Hospital and the Magee Women's Research Institute, and the Global Alliance to Prevent Prematurity and Still Birth	not stated	plasma	liquid chromatography mass spec	10-12 weeks	sPTB <35 weeks	case-control	USA	F13A, GBLN1, IC1, ITIH2, LCAT<br><br>IC1, LCAT, TRFE, ITIH4	y<br><br>y	panel of proteins revealed AUC 0.74, 0.77 in nulliparous women using a different panel	different multimarker panels were identified as being the highest performing for primipara vs multipara
58	American Journal of Obstetrics and Gynecology	Mclean et al.	1999	evaluate the ability of high plasma placental peptide CRH and AFP to prospectively discriminate pregnancies at high risk of preterm delivery	primarily anglo-saxon white, middle-class australians, median ga at preterm delivery 34 weeks	n=37 sPTB, n=860 tB	John Hunger Hospital	not stated	plasma	radioimmunoassay	17-30 weeks	sPTB <37 weeks	cohort	Australia	CRH<br>AFP	y<br>y	those with sPTB had higher concentration sof CRH and AFP than term. 3 marker combination predicted better than either alone	no significant difference between nulliparous and multiparous women or between pregnancies carrying a male fetus vs female fetus
59	Science	Ngo et al.	2018	develop a classifier to identify women who are at risk of preterm delivery	predominantly african american, all had a prior spontaneous PTB and received progesterone injections, mean age 25, mean BMI 30	n=8 sPTB, n=7 TB	Denmark, University of Pennsylvania and University of Alabama cohorts	not stated	blood	RNAseq then RT-PCR	12-37 weeks	sPTB <37 weeks	cohort	USA and Denmark	panel of 7 cfRNAs predicted sPTB: *CLCN4, DAPP1, PPBP, MAP3K7CL, MOB1B, RAB27B, RGS18*<br><br>38 cfRNAs were differentially expressed in sPTB compared to term - unclear what these are	u	top seven cfRNAs from the panel accuarately classified 6 of 8 preterm and misclassified 1/26 terms	na
60	EBioMedicine	Olsen SF et al.	2018	examine the association between plasma fatty acids quantiifed in pregnancy and subsequent risk of early PTB	primarily 21-45 years old, in a couple, non smokers, prepregnancy BMI 18.5-24.9, and own their own residence, 57% nulliparous	n=376 sPTB, n=348 TB	Danish National Birth Cohort	1996-2002	plasma	liquid chromatography mass spec	9 and 25 weeks	sPTB <34 weeks	case-control	Denmark	eicosapentaenoic acid + docosahexaenoic acid	y	risk of sPTB increases with lower EPA and DHA levels	na
61	Journal of Maternal-Fetal and Neonatal Medicine	Olsen RN et al.	2014	evalute whether maternal estriol concentrations from second trimester serum correlated with PTD	mean age 29, 20% of American Indians in the cohort had a preterm delivery, preterms were more likely to have higher gravidity than terms	n=7767	women who had second trimester maternal serum aneuploidy screening	1995-2010	serum	immunoassay	15-22 weeks	sPTB <37, 24-28, 28-32, 32-35 and 35-37 weeks	cohort	USA	uE3	y	elevated estriol was significantly associated with PTB and estriol level correlated with gestational age at delivery	estriol was more predictive of PTB at 32-34.9 weeks than at 24 27.9, 28-31.9 and 35-37 weeks
62	American Journal of Obstetrics and Gynecology	Parry et al.	2014	identify serum biomarkers of early sPTB using semi-quantitative proteomic analyses	women with previous sPTB, mean age 26, primarily caucasion or black or african american, mean BMI 26, approx 30% college educated, 31% gravidity=1	n=35 sPTB, n=35 TB	University of Alabama at Birmingham, University of Texas Medical Branch at Galveston and Unversity of Utah with history of at least one sPTB	not stated	serum	liquid chromatography mass spec and western blot	19-24 and 28-32 weeks	sPTB <34 and 34/27 weeks	case-control	USA	differentially expressed in term and preterms: cadherin-1, calreticulun precursor, serpinB7, proteasome subunit B type-5 isoform 3, glia-derived nexin isoform c prescursor, galectin-3 binding protein, peptidyprolyl isomerase 3, heat shock protein B1, thrombospondin-1 precursor, fibronectin-1, isoform 1 preprotein, zin-a2-glycoprotein, histidine-rish glycoprotein, pappalysin-1, carboxypeptidase N, serum amyloid P-component, apolipoprotein A-IV, apolipoprotein E, a2-antiplasmin isoform b, ficolin-3 isoform 1, corticosteroid binding globulin, kiniogen-1 isoform 1, hyalouronan-binding protein-2 isoform 1, plasminogen isoform 1, N-acetylmuramoyl-L-alanine amidase, IGFc-BP, pregnancy-zone protein, prothrombin, retinol binding protein-4, SHBG isoform 3, vitronectin, SERPING1<br><br>validated: SerpinB7	y<br><br>y	serpin B7 was 1.5fold higherin sPTB compared to controls and associated with shorter interval to delivery and lower gestational age at delivery	na
63	International Journal of Gynecology and Obstetrics	Paternoster et al.	2002	to identify predictive biochemical markers for sPTB	all caucasian, no risk factors for PTB, 68% nulliparas, mean age 33	n=2254	women receiving routine prenatal care in the Gynecology and Obstetrics Institute of Padova University	not stated	serum	chemiluminescent immunometric assay	24 weeks	sPTB <37 weeks	cohort	Italy	IL6<br>IL8<br>TNFa<br>ferritin<br><br>ferritin, cervical IL6, vaginal pH and cervical fFN	n<br>n<br>n<br>y<br><br>y	serum ferritin was higher in sPTB compared to term. Cervical markers were best marks for prediction	serum ferritin was predictive of PTB <35 weeks but not overall PTB or PTB <32 weeks
64	Journal of Obstetrics and Gynaecology of India	Patil et al.	2014	to study the pregnancy outcome in relation to the variations of PAPP-A level in the first trimester	patients registered to deliver at Bharati Hospital and Research Centre, Pune. No participant demographic information	n=524	patients receiving first rimester screening	not stated	serum	PerkinElmer lab (not described)	11-13 weeks	sPTB 32-36 and 28-32 weeks	cohort	India	PAPP-A	y	low PAPP-A is associated with high risk for preterm delivery, with low positive predictive value	na

65	British Journal of Obstetrics and Gynaecology	Petersen et al.	1992	determine whether high serum relaxin concentrations in the 30th week of pregnancy were associated with preterm labour	living in Aarhus town or certain designated areas around the town, mean age 29, rate of sPTB was 2.4%, majority primiparous,	n=32 sPTB, n=46 TB	Two antenatal clinics of the deparatment of Obstetrics and Gynecology University Hospital Aarhus	1988-1989	serum	ELISA	30 weeks	sPTB <37 weeks	case-control	Denmark	relaxin	y	high serum relaxin concentrations may be associated with preterm delivery	difference in relaxin levels between preterms and terms was more pronounced in primiparous women;<br>relaxin levels were higher in PPROM than in sPTD without PPROM (not signifiant)
66	Prenatal Diagnosis	Pihl et al.	2009	establish the first trimester serum levels of the proform of eosinophil major basic protein (proMBP) in pregnancies with adverse outcome, and to determine the screening performance using proMBP alone and in combination with other first trimester markers	all caucasian, median age 29, preterms were more likely than terms to be younger, smoke and to be nulliparous	n=88 sPTB, n=500 TB	enrolled in first trimester combine screening program for Down's syndrome	2005-2007	serum	immunofluorescence assay	TM1	sPTB <37	case-control	Denmark	proform of eosinophil major basic protein	y	proMBP was reduced in pregnancies with sPTB	proMBP was reduced in late sPTD(36-37 weeks) but not in earlier PTD
67	Prenatal Diagnosis	Pihl et al.	2009	establish the first trimester levels of pregnancy specific beta 1 glycoprotein (SP1) in pregnancies with adverse outcome and to determine the screening performance for adverse outcome using SP1 alone and in combination with other first trimester markers including proMBP and PAPP-A	all caucasian, median age 29, preterms were more likely than terms to be younger, smoke and to be nulliparous	n=88 sPTB, n=500 TB	enrolled in first trimester combine screening program for Down's syndrome	2005-2007	serum	immunofluorescence assay	TM1	sPTB <37	case-control	Denmark	pregnancy specific beta 1 glycoprotein	y	SPA was significnatly reduced in pregnancies with sPTB, only slightly improved prediction when combined with PAPP-A or proMBP	SP1 was lower in late sPTD (36-37 weeks) but not in earlier PTD
68	American Journal of Epidemiology	Pitiphat et al.	2005	examine the association of CRP with preterm delivery	primarily white or black, 16.7% had smoked in the 3 months before they knew they were pregnant, preterms were more likely to be heavier before conception and to have had a previous PTB and a gentiourinary infection during the current pregnancy	n=117 sPTB, n=117 TB	Project Viva	1999-2002	serum	immunoturbidimetric assay	5.3-19.3 weeks	sPTB <37 weeks	case-control	USA	CRP	y	no association between CRP thresholds and PTB, but very high levels of CRP were associated with increased risk of PTD	CRP levels were higher in women who had a previous PTD than those who had not and higher in PTD <34 weeks than PTD between 34 and 37 weeks
69	Prenatal Diagnosis	Poon et al.	2009	investigate the potential value of maternal serum MMP-9 in first trimester screening for preeclampsia and sPTB	primarily white, spontaneous conception, median age 32, approx half nulliparous, preterms were more likely to smoke, to have diabetes mellitus and to have had a previous sPTB	n=57 sPTB, n=569 TB	routine assessment of risk for chromosomal abnormalities by fetal nuchal translucency thickness and serum screens	not stated	serum	ELISA	11-13 weeks	sPTB <34 weeks	case-control	UK	MMP-9<br>TNFR1<br>PAPP-A	y<br>n<br>n	MMP-9 was higher at term than sPTB. Prediction was not improved with MMP-9 over history alone	na
70	Fetal Diagnosis and Therapy	Poon et al.	2013	examine the possible relationship between maternal and fetal characteristics and pregnancy outcomes on fetal and maternal cell free DNA in maternal plasma at 11-13 weeks gestation	median age 31.8, median weight 65 kg, median height 164 cm, 70% caucasian, 20% afro-caribbean, 6.2% smokers, 98% spontaneous conception	n=1949	prospective first trimester combine screening for aneuploidies and adverse outcomes	not stated	plasma	chromosome selective sequencing	11-13 weeks	sPTB <34 weeks	cohort	UK	[maternal cfDNA]<br>[fetal cfDNA]	n	cfDNA was not significantly altered in pregnancies complicated by sPTB	na
71	Biological Research for Nursing	Ruiz et al.	2002	investigate the relationships and predictability of CRH on gestational age at birth, develop a predictive model, examine differences in CRH by groups and examine proportions of cigarette smoking in groups that had high or low stress scores	predominantly medicaid clients, age range 18-40 years (49% 21-29 years), 25% less than high school education, 75% anglo-american, 20% hispanic, 60% married, mean ga at delivery was 40 weeks, higher gravidity = higher % of PTL	n=78	private practice of 2 obstetriciangynecologists in central Texas	not stated	plasma	radioimmunoassay	24 or 32 weeks	sPTB <37 weeks	cohort	USA	CRH	y	stress and CRH was weakly associated, ethnicity did not improve prediction	na?
72	American Journal of Obstetrics and Gynecology	Saade et al.	2016	develop and validate a mass spectrometry based serum test to predict sPTB in asymptomatic pregnancy women	mean age 28, median BMI 27, 70% white, 23% black or african american, approx 70% multigravida, 17% smoking, preterms were more likely to have had 1 or more previous PTDs and to have experienced bleeding after 12 weeks in the study pregnancy, controls in the overal validation cohort were more likely to be hispanic	n=5501	prospective Proteomic Assessment of Preterm Risk at 11 sites in USA	2011-2013	serum	multiple monitoring mass spec	17-28 weeks	sPTB <37 weeks	case-control	USA	IBP4/SHBG	y	IBP4/SHBG predictor AUC 0.75 sensitivity and specificity of 0.75 and 0.74. accuracy increased for early sPTB (<35 weeks)	predictive performance was improved by BMI stratification of >22 and <=37
73	Taiwanese Journal of Obstetrics and Gynaecology	Shin et al.	2016	investigate which ultrasound findings or serum biomarkers, including insulin like growth factor (IGF-1) and insuling like growth factor binding protein 1 and 3 (OGFBP1, IGBP3) in the first and early second trimesters are the best predictors for preterm delivery	median age 33, median height 161, median weight 55 kg, median BMI 22, approx half nulliparous	n=25 sPTB, n=48 TB	women receiving routine antenatal care at 11-18 weeks	2011-2013	serum	ELISA, immunoturbidimetric assay, bead based assay	11-18 weeks	sPTB 24-36 weeks	case-control	Korea	total cholesterol<br>HDL<br>estriol<br>AFP<br>inhibin A<br>hCG<br>IGF-1<br>IGFBP-1<br>IGFBP-3<br>CRP<br>procalcitonin<br>NGAL<br>PlGF<br>IL2<br>IL6<br>IL10<br>IL12<br>IL17<br>IFNy<br>TNFa	n<br>n<br>n<br>y<br>n<br>n<br>y<br>n<br>y<br>n<br>n<br>n<br>n<br>n<br>n<br>n<br>n<br>n<br>n<br>n	IGBP-3 and cervical length were significantly higher SPTB	na
74	American Journal of Obstetrics and Gynaecology	Sibai et al.	2005	examine the utility of a single second trimester plasma corticotropin releasin hormone measurement as a marker for preterm delivery in women at high risk for preterm delivery	women with at least 1 previous spontaneous PTD, 77.1% african american, mean age 25, preterms were more likely to have a higher number of previous sPTDs	n=173	NICHD multicenter placebo controlled trial designed to evaluate the role of 17 alpha hydroxyprogesterone caproate in the prevention of recurrent preterm birth	not stated	plasma	ELISA	16-20 weeks	sPTB <37 weeks	cohort	USA	CRH	n	no differences in CRH in those that delivered <37 weeks or <35 weeks compared to term	na
75	Obstetrics and Gynecology	Smith et al.	2007	estimate the relationship between maternal serum levels of placental growth factor (PIGF) and soluble fms like tyrosine kinase 1 (sFlt-1) in early pregnancy with the risk of subsequent adverse outcome	primarily white, non smokers, median age 29.5, approx half nulliparous,approx half married, median height 1.62 m, median BMI 23.7	n=65 early sPTB, n=227 late sPTB, n=937 TB	prospective cohort study of Down syndrome screening	1997-1999	serum	ELISA	10-14 weeks	sPTB 24-32 and 33-36 weeks	case-control	UK	sFlt-1<br>PlGF	y<br>n	higher levels of sFlt-1 were associated with reduced risk of extreme and moderate sPTB	na
76	International Journal of Epidemiology	Smith et al.	2006	determine which maternal and biochemical factors are associated with the relative risk of sPTB and whether the relative risks associated with these factors signfiicantly varied over the perid of 24-36 weeks gestation and whether these factors could yield clinically useful prediction of risk	all nulliparous, median age 26, median height 163, median BMI 23.5, 30% smokers, approx half married, preterms were more likely to be shorter, to smoke, and to have had a previous miscarriage or previous therapeutic abortion	n=84391	Scottish Morbidity Record from Scottish maternity hospitals	not stated	serum	maternal serum screen (secondary data)	TM2	sPTB 24-36 weeks	cohort	Scotland	AFP<br>hCG	y<br>y	risk of sPTB was positively associated with AFP. Risk of sPTB at 24-28 weeks was associated with hCG	high levels of hCG and age <20 were associated with extreme PTB but not mild or moderate PTB

77	Journal of Maternal-Fetal and Neonatal Medicine	Soni et al.	2018	evaluate relationship between first and second trimester maternal serum free B-hCG and the risk of sPTB	mean age 32, mean BMI 31, 63% caucasian, 10% african american, preterms were more likely to be older and african american and to have had a prior PTD	n=173 sPTB, n=704 TB	women evaluted at antepartum units and delivered at hospitals in the Northwell Health System	2011-2015	serum	PerkinElmer labs (not described)	11-13 weeks	sPTB <37 weeks with or without PPROM	case-control	USA	B-hCG	y	high maternal first and second trimester B-hCG was assocaited with low rates of PTB, but not associated with different likelihood of PTD	high B-hCG was more strongly associated with decreased odds of PTD in those without prior PTD than in those with prior PTD
78	Ultrasound in Obstetrics and Gynecology	Spencer et al.	2008	examine the clinical utility of the first trimester markers of aneuploidy in their ability to predict preterm delivery	primarily caucasian, mean age 31, mean weight 64 kg, approx 11% smokers, approx half nulliparous,	n=57422	patients that had undergone screening for trisomy 21 by fetal nuchal translucency thickness and maternal serum free B-hCG and PAPP-A	1998-2003	serum	kryptor analyzer	11-13 weeks	sPTB <37 weeks	cohort	UK	b-hCG<br>PAPP-A	n<br>y	low levels of PAPP-A are associated with increased risk of PTB	none
79	Fertility and Sterility	Stegmann et al.	2015	determine the association of PTB with antimullerian hormone levels both in isolation and in combination with other markers of the fetoplacental health commonly measured during integrated prenatal screening for aneuploidy	all white, mean age 28.8, mostly non smokers	n=112 sPTB, n=112 term	maternal fetal serum tissu ebank at the University of Iowa	2009-2010	serum	reprosource AMH assay	15-20 weeks	sPTB <37 weeks	case-control	USA	anti-mullerian hormone<br>AMH adjusted for AFP	n<br>y	second trimester AMH was not associated with PTB, but was assocaited with adjusted for AFP and maternal weight change between the 1st and 2nd trimesters	no fetal sex specific interactions were observed; addition of history of PTB did not affect models
80	Cell Reports Medicine	Tarca et al.	2021	conduct a multiomics study to develop predictive models of SPTB	transcriptomics: mean age 25, 94% african american, 29% nulliparous, preterms were more likely to have a history of PTB<br>proteomics: mean age 24, 91% african american, %24 nulliparous, preterms were more likely to have a history of PTB	n=34 sPTB, n=65 TB (transcriptome), n=62 sPTB, n=39 TB (proteome)	longitudinal study at the Center for Advanced Obstetrical Care and Research of the Perinatology Research Brance, the NICHD/NIH/DHHS, the Detroit Medical Cewnter, the Wayne State University School of medicine	not stated	whole blood	microarray (transcriptome)<br>SOMAmer aptamer based (proteomic)	<33 weeks	sPTB <37 weeks	case-control	USA/Canada	50 genes used to predict (but not listed)<br><br>50 proteins used to predict (but not all listed): AGER, PDPK1, LAG3, HAVR2, IL6, FCER2, cADM1, PLAUR, IMPDH2, PRDX6, PA2G4, F2, PPIE, GDI2, NAPA, PDPK1, MFGE8, ANGPT1, CAM2A	y	RNA is differentially expressed in PPROM (same across two cohorts). Proteomic profiles predict sPTB better than gene expression	addition of cases with PPROM to those with sPTD did not affect predictive performance of proteomics model
81	Journal of Pregnancy	Tripathi et al.	2014	evaluate the role of biomarkers and cervical length in prediction of preterm labour in asymptomatic low risk women in an Indian population	women at low risk of PTB, mean BMI 22, nulliparity around %40, primarily non smokers	n=137 sPTB, n=137 TB	tertiary care teaching hospital	not stated	serum	ELISA	24-27 weeks	sPTB <35 weeks	case-control	India	G-CSF<br>AFP<br>ferritin<br>IL6<br>iron<br>alkaline phosphatase<br>cervical IGFBP-1, AFP, G-CSF, alkaline phosphatase, IL-6, cervical length	n<br>n<br>y<br>n<br>n<br>y<br>y	alkaline phosphatase and ferritin was associated with preterm delivery but had poor predictive value	na
82	American Journal of Obstetrics and Gynaecology	Vogel et al.	2006	evaluate the relationship between serum concentrations of relaxin and sCD163 with cervical length and preterm delivery in women with previous sPTB	women with at least 1 late spontaneous miscarriage or early sPTB between 16 and 30 weeks, median age 24, median prepregnancy weight 63 kg, median height 165, median BMI 24, 84% black race, 26% greater than 1 previous PTD, 13% smoking in pregnancy, 87% unmarried, participants with PTD <37 weeks were more likely to be black	n=61	University of Alabama at Birmingham	1995-1996	serum	ELISA	12-25 weeks	sPTB <37, <35, <32 weeks with or without PPROM	cohort	Denmark	relaxin<br>scd163	n	relaxin and sCD163 were not correlated with sPTB	C
83	Journal of Reproductive Immunology	Vogel et al.	2007	analyze the association between serum and cervicovaginal inflammatory markers and recurrent sPTB in women with prior early sPTB	women with at least 1 prior spontaneous PTB between 16 and 30 weeks, median age 24, median prepregnancy weight 63 kg, median height 165 cm, median BMI 24, 84% african american, 26% greater than 1 previous PTD, 13% smoking in pregnancy, 87% unmarried	n=62	University of Alabama at Birmingham	not stated	serum	multiplex bead based assay	12-25 weeks	sPTB <35, <37 weeks	cohort	Denmark	IL1B<br>IL2<br>IL4<br>IL5<br>IL6<br>IL8<br>IL10<br>IL12<br>IL17<br>IL18<br>TNFa<br>MMP-9<br>TGF-B1<br>sTNF-R1<br>GM-CSF<br>sIL-6Ra<br>TREM-1<br>TNFa, cervical IL6-Ra and cervical length	y<br>y<br>n<br>y<br>y<br>y<br>n<br>y<br>n<br>y<br>y<br>n<br>y<br>y<br>y<br>n<br>y<br>y	serum TNFa, cervicovaginal sIL6Ra and cervical length predicted 69% of sPTB, 5% false positive, multiple inflammatory markers were associated with sPTB	stratifying data based on cervical length did not significantly affect prediction results: inflammatory markers were predictors of PTB in women with long cervical length
84	Journal of Women's Health	Whitcomb et al.	2009	evaluate the association between maternal serum G-CSF in the first and second trimstesters and PTB	mean age 25, 43% smoking at time of presentation for prenatal care, 20% infection in pregnancy, 71% white, 24% black, preterms were more likely to be non white, to smoke, and to have an infection in pregnancy, but not significant	n=31 sPTB, n=394 TB	Collaborative Perinatal Project	1959-1974	serum	multiplex fluorokine (ELISA based)	first and second trimesters	sPTB <37 weeks	case-control	USA	G-CSF	y	G-CSF was significantly assocaited with gestational age at birth and PTB	estimates were not substantially altered when considering previous PTB as a risk factor. Mean G-CSF was higher among PTB <33 weeks compared to all PTB
85	PloS ONE	Winger et al.	2020	predict sPTB among pregnant women in an African American population using first trimester peripheral blood maternal immune cell microRNA	black women with singleton pregnancies and maternal BMI <30, mean age 23, mean BMI 23, 46% nulliparous, 11% prior PTB, 48% prior spontaneous abortion, 15% smoking, preterms were more likely to have had a prior PTB	n=18 sPTB, n=139 TB	Center for Advanced Obstetrical Care and Research of the Perinatology Research Branch	2006-2016	blood	rt-qPCR	6-12 weeks	sPTB <35 weeks	case-control	USA	hsa_miR-221_3p, hsa-miR,33a_5p, has-miR_6752_3p, has-miR_1244, msa-miR_148a_3p<br>has_miR_1_3p, has_miR_1_3p, has_miR_1267, has_miR_223_5p, has_miR_199b_5p, has_miR_133b_msa_miR_144_3P, hsa_miR_4485_5p, has_miR_340_5p, has_miR_132_3p, has_miR_132_3p, has_miR_219_5p<br><br>hsa_miR_424_5p, hsa_miR_221_5p, hsa_miR_1237_3p, hsa_miR_199a, 5p, hsa_miR155_5p, hsa_miR301a_3p, hsa_miR_575, hsa_miR_6889_3p, hsa_miR_30e_5p, hsa_miR146a_5-, hsa_miR_146a_5p, hsa_miR_30e_3p, hsa_miR_551b_3p, hsa_miR_582_5p, hsa_miR_671_5p, hsa_miR_7_5p, hsa_miR_1229_5p, hsa_miR_6819_3p, hsa_miR_196a_5p, hsa_miR_210_5p, hsa_miR_1238_3p, hsa_miR_6737_3p, hsa_miR_193a_3p, hsa_miR_223_3p, hsa_miR_181a_5p, hsa_miR_210_3p, hsa_miR_24_1_5p, hsa_miR_6757_3p, hsa_miR_513_5p, hsa_miR_16_5p	y<br><br>n	miRNA panel predicted in test set with AUC 0.80, sensitivity 0.89 and specificity 0.71	na
86	PLoS ONE	Wommack et al.	2018	determine association between microRNA clusters and PTB and infant birth outcomes	Hispanic women between the ages of 18 and 39, born in the US or had lived in the US for 10 or more years, covered by self-pay, state funded or private insurance, no use of steroids or antidepressants 1 month prior to enrollment, mean age 26, mean prepregnancy BMI 25, 5% cotine positive, 24% history of PTB	n=21 sPTB, n=21 TB	Hispanic women recruited at two obstetrical clinics in Houston metropolitan area	2008-2012	plasma	miRCURY LNA universal RT microRNA PCR panel	22-24 weeks	sPTB <37 weeks	case-control	USA	has_miR_127-3p<br>has_miR_136_5p<br>has_miR_543<br><br>c19mc correlation with c14mc miRNA clusters<br>c14mc cluster<br>c19mc	y<br>y<br>y<br><br>y<br>y<br>y	groups of miRNAs from common chromosal clusters, not individual miRNAs are associated with length of gestation and sPTB	na
87	Reproductive Sciences	Zhou et al.	2020	examine sPTB in relation to maternal b lood mRNA levels of previously identified genes associated with sPTB	primarily caucasian, mean age 31,mean BMI 25, approx 15% smoking during pregnancy, approx half nulliparous	n=51 sPTB, n=106 TB	public gene expression dataset from the All Our Families cohort in Calgary, Canada	2008-2010	blood	Affymetrix microarray	TM1 and TM2	sPTB <37 weeks	case-control	Canada	EBF1<br>EEFSEC<br>AGTR2<br>WNT4<br>ADCY5<br>RAP2C	y<br>n<br>n<br>n<br>n<br>n	women in the lowest quartile of EBF1 the odds of sPTB was 2.86, no other candidate genes were significantly associated with sPTB	similar predictions for sPTB participants with and without PPROM
88	Clinica Chimica Acta	Zhu et al.	2018	assess the relationship between maternal early pregnancy plasma concentrations of macrophage inhibitor factor (MIF) and sPTB	no abortions or fetal demise<br>all primigravidas, primarily Han Chinese, median age 28, median prepregnancy BMI 24, 11% smoking at study entry, 7% alcohol consumption in first trimester, 94% married, 20% high school or less education, 12% gentourinary infection during pregnancy, 49% "middle" socio-professional category, preterms were more likely to be older, have a higher prepregnancy BMI, smoke, and have lower phsyical activity levels	n=596	first time pregnancies attending first prenatal visit	2016	plasma	ELISA	<14 weeks	sPTB <37 weeks	cohort	China	MIF<br>CRP<br>IL6	y<br>y<br>y	MIF was higher in sPTB compared to term, 1ng/nL increase assocaited with increased 12% odds of sPTB and improved discriminatory over BMI, CRP and IL-6	na
