# Supplementary material for: Is there a maternal blood biomarker that can predict spontaneous preterm birth prior to labour onset? A systematic review
Source: PLoS One. 2022 Apr 4;17(4):e0265853. doi: 10.1371/journal.pone.0265853 (PMC8979439; doi:10.1371/journal.pone.0265853)
Supplement: S4 File — (DOCX) [file pone.0265853.s004.docx]

**Table 1. Maternal blood biomarkers of sPTB**

Biomarkers associated with or predictive of sPTB prior to labour symptoms in at least one study.

| **Biomarker** | **Description** | **Reference** |
| --- | --- | --- |
| 11(12)-EET | 11,12-epoxyeicosatrienoic acid | [16] |
| 11,12-DHET | 11,12-dihydroxy-5Z,8Z,14Z-eicosatrienoic acid | [16] |
| 12(1)-EpoME | vernolic acid | [16] |
| 12,13-DiHOME | (9Z)-12,13-dihydroxyoctadec-9-enoic acid | [16] |
| 12-HETE | 12-hydroxyeicosatetraenoic acid | [16] |
| 12-oxoETE | oxoicosatetraenoic acid | [16] |
| 13,14-DHK-PGD2 | 13,14-dihydro-15-keto-prostaglandin D2 | [16] |
| 13,14DHK-PGF2a | 13,14-dihydro-15-keto-prostaglandin F2a | [16] |
| 13-oxoODE | 13-oxooctadecadienoic acid | [16] |
| 15DO12,14-PGJ2 | 15-deoxy-delta-12,14-prostagladin J2 | [16] |
| 15-HETE | 15-hydroxyeicosatetraenoic acid | [16] |
| 5(6)-EET | 5,6-epoxy-8Z,11Z,14Z-eicosatrienoic acid | [16] |
| 5-HETE | 5-hydroxyeicosatetraenoic acid | [16] |
| 5-oxoETE | 5-oxo-eicosatetraenoic acid | [16] |
| 8(9)-Eet | 8,9-epoxyeicosatrienoic acid | [16] |
| 8,9-DHET | 8,9-dihydroxy-5Z,11z,14Z-eicosatrienoic acid | [16] |
| 8-HETE | hydroxyeicosatetraenoic acid | [16] |
| 9(1)-Epo-ME | erythropoietin | [16] |
| 9,10-DiHOME | 9,10-dihydroxy-12-octadecenoic acid | [16] |
| 9-oxoODE | 9-oxooctadecadienoic acid | [16] |
| A1BG | alpha-1-B glycoprotein | [32] |
| A2JA19 | anti-mucin1 light chain variable region | [32] |
| A2M | alpha-2-macroglobulin | [24, 32] |
| AA | arachidonic acid | [16] |
| ACAP2 | ArfGAP with coiled-coil, ankyrin repeat and PH domains 2 | [38] |
| ADAM12 | ADAM metallopeptidase domain 12 | [20] |
| ADAMTS15 | ADAM metallopeptidase with thrombospondin type 1 motif 15 | [53] |
| AFP | alpha fetoprotein | [14, 19, 34, 40, 43, 44, 45, 58, 73, 76, 81] |
| AGE | advanced glycation end products | [47] |
| AGER | advanced glycosylation end-product specific receptor | [46, 80] |
| ALB | albumin | [32] |
| ALPL | alkaline phosphatase, biomineralization associated | [34, 81] |
| AMBP | alpha-1-microglobulin/bikunin precursor | [24] |
| ANGPT1 | angiopoietin 1 | [56, 80] |
| ANGPT2 | angiopoietin 2 | [53, 56] |
| ANGPTL3 | angiopoietin like 3 | [56] |
| APCS | amyloid P component, serum | [53, 62] |
| APOA1 | apolipoprotein A1 | [24] |
| APOA4 | apolipoprotein A4 | [62] |
| APOD | apolipoprotein D | [32] |
| APOE | apolipoprotein E | [62] |
| APOL1 | apolipoprotein L1 | [24, 32] |
| APOM | apolipoprotein M | [24] |
| AZGP1 | alpha-2-glycoprotein 1, zinc-binding | [32, 62] |
| AZU1 | azurocidin 1 | [29] |
| B2M | beta-2-microglobulin | [34, 55] |
| C1R | complement C1r | [32] |
| C3 | complement C3 | [32] |
| C4A | complement C4A (Rodgers blood group) | [32] |
| C5 | complement C5 | [53, 56] |
| C8A | complement C8 alpha chain | [24] |
| C9 | complement C9 | [24] |
| CADM1 | cell adhesion molecule 1 | [80] |
| CALR | calreticulin | [62] |
| CAMK2A | calcium/calmodulin dependent protein kinase II alpha | [80] |
| CCL11 | C-C motif chemokine ligand 11 | [39, 46] |
| CCL2 | C-C motif chemokine ligand 2 | [39, 46] |
| CCL4 | C-C motif chemokine ligand 4 | [39, 46] |
| CCL5 | C-C motif chemokine ligand 5 | [39, 46, 56] |
| CCL7 | C-C motif chemokine ligand 7 | [39, 46] |
| CD5L | CD5 molecule like | [24] |
| CDH1 | cadherin 1 | [62] |
| CDH3 | cadherin 3 | [53] |
| CFB | complement factor B | [53] |
| CFH | complement factor H | [32, 53] |
| CGB3 | chorionic gonadotropin subunit beta 3 | [14, 40, 43, 44, 45, 73, 76] |
| CHI3L1 | chitinase 3 like 1 | [56] |
| CHL1 | cell adhesion molecule L1 like | [55] |
| CLCN4 | chloride voltage-gated channel 4 | [59] |
| Co | cobalt | [15] |
| CPN1 | carboxypeptidase N subunit 1 | [24, 62] |
| CRH | corticotropin releasing hormone | [31, 34, 52, 58, 71, 74] |
| CRP | C-reactive protein | [16, 17, 23, 25, 33, 34, 41, 48, 56, 68, 73, 88] |
| CSF1 | colony stimulating factor 1 | [46] |
| CSF2 | colony stimulating factor 2 | [27, 39, 46, 83] |
| CSF3 | colony stimulating factor 3 | [34, 35, 39, 46, 81, 84] |
| CST13P | cystatin 13, pseudogene | [38] |
| CTSA | cathepsin A | [53] |
| CTSZ | cathepsin Z | [53] |
| CXCL5 | C-X-C motif chemokine ligand 5 | [46] |
| CXCL8 | C-X-C motif chemokine ligand 8 | [39, 46, 63, 83] |
| CXCL9 | C-X-C motif chemokine ligand 9 | [46] |
| CXCR3 | C-X-C motif chemokine receptor 3 | [55] |
| CYC1 | cytochrome c1 | [55] |
| DAPP1 | dual adaptor of phosphotyrosine and 3-phosphoinositides 1 | [59] |
| DDAH2 | dimethylarginine dimethylaminohydrolase 2 | [55] |
| DEFB1 | defensin beta 1 | [31, 34] |
| DHA | docosahexaenoic acid | [16, 60] |
| DKKL1 | dickkopf like acrosomal protein 1 | [53] |
| DUSP1 | dual specificity phosphatase 1 | [55] |
| EBF1 | EBF transcription factor 1 | [87] |
| EFHD2 | EF-hand domain family member D2 | [38] |
| ENG | endoglin | [56] |
| EPA | eicosapentaenoic acid | [16, 60] |
| ERVMER34-1 | endogenous retrovirus group MER34 member 1 | [24] |
| F13A1 | coagulation factor XIII A chain | [24, 57] |
| F13B | coagulation factor XIII B chain | [24] |
| F2 | coagulation factor II, thrombin | [62, 80] |
| F9 | coagulation factor IX | [53] |
| FASLG | Fas ligand | [46] |
| FBLN1 | fibulin 1 | [24, 57] |
| FCER2 | Fc fragment of IgE receptor II | [80] |
| FCGBP | Fc fragment of IgG binding protein | [62] |
| FCN3 | ficolin 3 | [53, 62] |
| FGF2 | fibroblast growth factor 2 | [39, 46] |
| FLT1 | fms related receptor tyrosine kinase 1 | [16, 56, 75] |
| FLT4 | fms related receptor tyrosine kinase 4 | [46] |
| FN1 | fibronectin 1 | [53, 62] |
| FTH1 | ferritin heavy chain 1 | [12, 21, 31, 34, 48, 63, 81] |
| GC | GC vitamin D binding protein | [24] |
| GDI2 | GDP dissociation inhibitor 2 | [80] |
| GHRHR | growth hormone releasing hormone receptor | [53] |
| GLA | galactosidase alpha | [55] |
| HABP2 | hyaluronan binding protein 2 | [62] |
| HAVCR2 | hepatitis A virus cellular receptor 2 | [80] |
| HBA1 | hemoglobin subunit alpha 1 | [12, 54] |
| HBG1 | hemoglobin subunit gamma 1 | [20, 30, 49, 50, 77, 78] |
| HDL | high density lipoprotein | [14, 25, 73] |
| hematocrit | volume percentage red blood cells | [54] |
| HP | haptoglobin | [24] |
| HPX | hemopexin | [32, 53] |
| HRG | histidine rich glycoprotein | [62] |
| HSP90AB1 | heat shock protein 90 alpha family class B member 1 | [55] |
| HSPB1 | heat shock protein family B (small) member 1 | [62] |
| ICAM1 | intercellular adhesion molecule 1 | [34, 46, 56] |
| IFNB1 | interferon beta 1 | [46] |
| IFNG | interferon gamma | [27, 39, 46, 73] |
| IGFBP2 | insulin like growth factor binding protein 2 | [53] |
| IGFBP3 | insulin like growth factor binding protein 3 | [73] |
| IGFBP4 | insulin like growth factor binding protein 4 | [22, 72] |
| IGHA1 | immunoglobulin heavy constant alpha 1 | [32] |
| IGHM | immunoglobulin heavy constant mu | [32] |
| IL10 | interleukin 10 | [16, 33, 34, 39, 46, 73, 83] |
| IL10RA | interleukin 10 receptor subunit alpha | [55] |
| IL12A | interleukin 12A | [39, 46, 73, 83] |
| IL13 | interleukin 13 | [39, 46] |
| IL17A | interleukin 17A | [39, 46, 73, 83] |
| IL17F | interleukin 17F | [46] |
| IL18 | interleukin 18 | [39, 83] |
| IL18BP | interleukin 18 binding protein | [56] |
| IL1B | interleukin 1 beta | [16, 33, 39, 46, 56, 83] |
| IL1R2 | interleukin 1 receptor type 2 | [46] |
| IL2 | interleukin 2 | [27, 39, 46, 73, 83] |
| IL4 | interleukin 4 | [39, 46, 83] |
| IL4R | interleukin 4 receptor | [46] |
| IL5 | interleukin 5 | [39, 46, 83] |
| IL6 | interleukin 6 | [16, 28, 33, 34, 39, 46, 63, 73, 80, 81, 83] |
| IL6ST | interleukin 6 signal transducer | [46] |
| ILC2 | type 2 innate lymphoid cells | [13] |
| ILC3 | type 3 innate lymphoid cells | [13] |
| IMPDH2 | inosine monophosphate dehydrogenase 2 | [80] |
| INHBA | inhibin subunit beta A | [14, 20, 34, 40, 44, 45, 73] |
| ITIH1 | inter-alpha-trypsin inhibitor heavy chain 1 | [24] |
| ITIH2 | inter-alpha-trypsin inhibitor heavy chain 2 | [24, 57] |
| ITIH4 | inter-alpha-trypsin inhibitor heavy chain 4 | [24, 31, 57] |
| JAG1 | jagged canonical Notch ligand 1 | [53] |
| KDR | kinase insert domain receptor | [46, 53] |
| KITLG | KIT ligand | [46] |
| KLKB1 | kallikrein B1 | [24] |
| KNG1 | kininogen 1 | [24, 62] |
| LA | linoleic acid | [16] |
| LAG3 | lymphocyte activating 3 | [80] |
| LCAT | lecithin-cholesterol acyltransferase | [24, 39] |
| LEP | leptin | [46, 53, 56] |
| LGALS13 | galectin 13 | [20] |
| LGALS3BP | galectin 3 binding protein | [62] |
| LMLN2 | leishmanolysin like peptidase 2 | [38] |
| LTB4 | leukotriene B4 | [16] |
| LTC4-ME | N-methyl-leukotriene C4 | [16] |
| LTD4 | leukotriene D4 | [16] |
| LTE4 | leukotriene E4 | [16] |
| LTF | lactotransferrin | [31, 34] |
| lymphocyte | lymphocyte count | [54] |
| MAP2K2 | mitogen-activated protein kinase kinase 2 | [53] |
| MAP2K4 | mitogen-activated protein kinase kinase 4 | [53] |
| MAP3K7CL | MAP3K7 C-terminal like | [59] |
| MAPKAPK3 | MAPK activated protein kinase 3 | [53] |
| MBL2 | mannose binding protein C | [24] |
| MFGE8 | milk fat globule EGF and factor V/VIII domain containing | [80] |
| MIF | macrophage migration inhibitory factor | [39, 88] |
| MIR1-1 | microRNA 1 | [85] |
| MIR1244 | microRNA 1244 | [85] |
| MIR1267 | microRNA 1267 | [85] |
| MIR127 | microRNA 127 | [86] |
| MIR132 | microRNA 132 | [85] |
| MIR133 | microRNA 133 | [85] |
| MIR136 | microRNA 136 | [86] |
| MIR144 | microRNA 144 | [85] |
| MIR148 | microRNA 148 | [85] |
| MIR199 | microRNA 199 | [85] |
| MIR219 | microRNA 219 | [85] |
| MIR221 | microRNA 221 | [85] |
| MIR223 | microRNA 223 | [85] |
| MIR33A | microRNA 33A | [85] |
| MIR340 | microRNA 340 | [85] |
| MIR3691 | microRNA 3691 | [38] |
| MIR4485 | microRNA 4485 | [85] |
| MIR543 | microRNA 543 | [86] |
| MIR6572 | microRNA 6572 | [85] |
| MMP2 | matrix metallopeptidase 2 | [53] |
| MMP9 | matrix metallopeptidase 9 | [69, 83] |
| MOB1B | MOB kinase activator 1B | [59] |
| MSRA | methionine sulfoxide reductase A | [55] |
| NAPA | NSF attachment protein alpha | [80] |
| NCF1 | neutrophil cytosolic factor 1 | [55] |
| NCOA2 | nuclear receptor coactivator 2 | [55] |
| NEFA | non-esterified fatty acid | [25] |
| NGF | nerve growth factor | [46] |
| NOTCH3 | notch receptor 3 | [53] |
| PA2G4 | proliferation-associated 2G4 | [80] |
| PAPPA | pappalysin 1 | [14, 20, 30, 36, 42, 44, 45, 49, 50, 51, 53, 62, 64, 69, 78] |
| Pb | lead | [15] |
| PDE2A | phosphodiesterase 2A | [53] |
| PDGFB | platelet derived growth factor subunit B | [39, 46] |
| PDPK1 | 3-phosphoinositide dependent protein kinase 1 | [80] |
| PECAM1 | platelet and endothelial cell adhesion molecule 1 | [53] |
| PGB2 | prostaglandin B2 | [16] |
| PGE2 | prostaglandin E2 | [16] |
| PGF | placental growth factor | [16, 31, 31, 56, 73, 75] |
| PGJ2 | prostaglandin J2 | [16] |
| PGRP2 | peptidoglycan recognition protein 2 | [24, 62] |
| PIGR | polymeric immunoglobulin receptor | [53] |
| platelet | mean platelet volume | [54] |
| PLAUR | plasminogen activator, urokinase receptor | [80] |
| PLG | plasminogen | [62] |
| PPBP | pro-platelet basic protein | [59] |
| PPIE | peptidylprolyl isomerase E | [80] |
| PPIL3 | peptidylprolyl isomerase like 3 | [62] |
| PPP3CA | protein phosphatase 3 catalytic subunit alpha | [55] |
| PPP3R1 | protein phosphatase 3 regulatory subunit B, alpha | [53] |
| PRDX6 | peroxiredoxin 6 | [80] |
| PREX1 | phosphatidylinositol-3,4,5-trisphosphate dependent Rac exchange factor 1 | [55] |
| PRG2 | proteoglycan 2, pro eosinophil major basic protein | [66] |
| PROK1 | prokineticin 1 | [42] |
| PRSS3 | serine protease 3 | [24] |
| PSG1 | pregnancy specific beta-1-glycoprotein 1 | [67] |
| PSMB5 | proteasome 20S subunit beta 5 | [62] |
| PZP | PZP alpha-2-macroglobulin like | [62] |
| RAB27B | RAB27B, member RAS oncogene family | [62] |
| RBP4 | retinol binding protein 4 | [62] |
| RET | ret proto-oncogene | [53] |
| RETN | resistin | [46] |
| RGS18 | regulator of G protein signaling 18 | [59] |
| RLN1 | relaxin 1 | [34, 65, 82] |
| RNF7 | ring finger protein 7 | [55] |
| RUNX3 | RUNX family transcription factor 3 | [55] |
| RVD1 | resolvin D1 | [16] |
| RVD2 | resolvin D2 | [16] |
| SDHA | succinate dehydrogenase complex flavoprotein subunit A | [55] |
| SERPINA1 | serpin family A member 1 | [32] |
| SERPINA3 | serpin family A member 3 | [24] |
| SERPINA6 | serpin family A member 6 | [62] |
| SERPINA7 | serpin family A member 7 | [24] |
| SERPINB7 | serpin family B member 7 | [62] |
| SERPINC1 | serpin family C member 1 | [32] |
| SERPINE1 | serpin family E member 1 | [46] |
| SERPINE2 | serpin family E member 2 | [62] |
| SERPINF2 | serpin family F member 2 | [62] |
| SERPING1 | serpin family G member 1 | [24, 57, 62] |
| SH3PXD2B | SH3 and PX domains 2B | [38] |
| SHBG | sex hormone binding globulin | [22, 62, 72] |
| TAT | tyrosine aminotransferase | [31, 37] |
| TBX21 | T-box transcription factor 21 | [38] |
| TF | transferrin | [32, 57] |
| TGFB1 | transforming growth factor beta 1 | [46, 83] |
| THBS1 | thrombospondin 1 | [62] |
| TLR2 | toll like receptor 2 | [53, 55] |
| TLR4 | toll like receptor 4 | [55] |
| TNF | tumor necrosis factor | [16, 28, 33, 39, 46, 63, 73, 83] |
| TNFRSF1A | TNF receptor superfamily member 1A | [31, 69, 83] |
| TNFRSF1B | TNF receptor superfamily member 1B | [46, 56] |
| TNFSF10 | TNF superfamily member 10 | [39, 46] |
| TREM1 | triggering receptor expressed on myeloid cells 1 | [85] |
| TTR | transthyretin | [24] |
| uE3 | unconjugated estriol | [14, 40, 43, 44, 45, 61, 73] |
| VTN | vitronectin | [62] |
| Zn | microRNA | [15] |
| ZNF324 | zinc finger protein 324 | [38] |
