# Supplementary material for: Is there a maternal blood biomarker that can predict spontaneous preterm birth prior to labour onset? A systematic review
Source: PLoS One. 2022 Apr 4;17(4):e0265853. doi: 10.1371/journal.pone.0265853 (PMC8979439; doi:10.1371/journal.pone.0265853)
Supplement: S5 File — (DOCX) [file pone.0265853.s005.docx]

**Table 1. Odds Ratios of sPTB Against Maternal Blood Levels of Maternal Serum Screen Markers**

| **Biomarker** | **Study** | **Method** | **Tissue** | **Sample Size** | **Collection (weeks)** | **Outcome**  **(weeks)** | **OR (95% CI)** | **Cut-off** |
| --- | --- | --- | --- | --- | --- | --- | --- | --- |
| PAPP-A | Alleman et al 2014 | serum screen | serum | n=2499 TB  n=153 sPTB | 11-13 | <37 | ns |  |
|  | Beta et al 2011 | serum screen | serum | n=33017 TB  n=353 sPTB | 11-13 | <34 | **s but no OR** |  |
|  | El-Achi 2020 | serum screen | serum | n=10,397  n=144 PPROM | 11-13 | <37 | 0.4 (0.2-0.8) | >2 MoM |
|  | Gupta et al 2015 | serum screen | serum | n=298 TB  n=32 sPTB | 9-13 | <37 | **2.848 (0.982-8.266)** |  |
|  | Inan et al 2018 | immunoassay | serum | n=102 TB  n=16 sPTB | 11-13 | <37 | ns |  |
|  | Jelliffe-Pawlowski et al 2013 | serum screen | serum | n=1725 TB  n=345 sPTB | 10-13 | <30 | **2.1(1.3-3.5)** | 5th centile |
|  | Jelliffe-Pawlowski et al 2015 | serum screen | serum | n= 784,064 TB  n= 30,579 sPTB | 10-13 | <31 | **2.2(1.6-3.0)** | 5th centile |
|  |  |  |  |  |  | 32-36 | **1.7 (1.5-1.9)** |  |
|  | Kirkegaard et al 2010 | serum screen | serum | n=9100 TB  n=350 sPTB | 8-13 | <37 | **1.9 (1.0-3.6)** | <0.3 MoM |
|  |  |  |  |  |  | <34 | **2.4 (0.9-6.9)** |  |
|  | Kirkegaard et al 2011 | serum screen | serum | n=9100 TB  n=350 sPTB | 8-13 | <37 | **1.95(1.39-2.73)** | <0.4 MoM |
|  | Kwik et al 2003 | ELISA | serum | n=731 TB  n=96 sPTB | 11-13 | <37 | **s but no OR** |  |
|  | Patil et al 2013 | serum screen | serum | n=506 TB  n=18 sPTB | 11-13 | <37 | **s but no OR** | <0.5 MoM |
|  | Pihl et al 2009 | serum screen | serum | n=500 TB  n=88 sPTB | 8-13 | <37 | ns |  |
|  | Poon et al 2009 | serum screen | serum | n=569 TB  n=57 sPTB | 11-13 | <34 | ns |  |
|  | Poon et al 2013 | serum screen | serum | n=1919 TB  n=20 sPTB | 11-13 | <34 | ns |  |
|  | Pummara et al 2016 | serum screen | serum | n=2665 TB  n=270 sPTB | 11-13 | <37 | **2.366 (1.799-3.110)** | 10th centile |
|  |  |  |  | n=2665 TB  n=126 sPTB |  | <34 | **3.785 (2.620-5.469)** |  |
|  |  |  |  | n=2665 TB  n=99 sPTB |  | <32 | **5.408(3.654-8.003)** |  |
|  | Spencer et al 2008 | serum screen | serum | n=50,530 TB  n=3132 sPTB | 11-13 | <37 | **1.92** | 5th centile |
|  |  |  |  | n=50,530 TB  n=1060 sPTB |  | <34 | **2.35** |  |
| hCG | Alleman et al 2014 | serum screen | serum | n=2499 TB  n=153 sPTB | 11-13 | <37 | ns |  |
|  |  |  |  |  | 15-24 |  |  |  |
|  | Beta et al 2011 | serum screen | serum | n=33017 TB  n=353 sPTB | 11-13 | <34 | ns |  |
|  | El-Achi 2020 | serum screen | serum | n=10,397  n=144 PPROM | 11-13 | <37 | 0.9 (0.5-1.7) | >2 MoM |
|  | Jelliffe-Pawlowski et al 2010 | serum screen | serum | n=94,820 TB  n=1116 sPTB | 15-20 | <32 | 1.1 (0.8-1.4) | <0.5 MoM |
|  |  |  |  | n=94,820 TB  n=6933 sPTB |  | 33-36 | **1.1 (1.1-1.3)** |  |
|  |  |  |  | n=94,820 TB  n=8049 sPTB |  | <37 | **1.1 (1.0-1.2)** |  |
|  | Jelliffe-Pawlowski et al 2013 | serum screen | serum | n=1725 TB  n=345 sPTB | 11-13 | <30 | 0.5 (0.3-1.2) | 5^th^ centile |
|  |  |  |  |  | 15-20 |  | 0.5 (0.2-1.0) |  |
|  | Jelliffe-Pawlowski et al 2015 | serum screen | serum | n=784,064 TB, n=30,579 sPTB | 10-13 | <31 | 1.3 (0.9-2.0) | 5^th^ centile |
|  |  |  |  |  |  | 32-36 | **1.3 (1.1-1.5)** |  |
|  |  |  |  |  | 15-20 | <31 | 0.8 (0.4-1.3) |  |
|  |  |  |  |  |  | 32-36 | 1.1 (0.9-1.3) |  |
|  | Kirkegaard et al 2010 | serum screen | serum | n=9100 TB  n=350 sPTB | 8-13 | <37 | **2.0 (1.4-2.8)** | <0.5 MoM |
|  |  |  |  |  |  | <34 | 1.4 (0.7-2.8) |  |
|  | Kirkegaard et al 2011 | serum screen | serum | n=9100 TB  n=350 sPTB | 8-13 | <37 | 1.50 (0.95-2.38) | <0.4 MoM |
|  | Poon et al 2013 | serum screen | serum | n=1919 TB  n=20 sPTB | 11-13 | <34 | ns |  |
|  | Smith et al 2006 | serum screen | serum | n=79,116 TB  n=3017 sPTB | 15-24 | <37 | 1.05 (0.94-1.17) | 20^th^ centile |
|  | Soni et al 2018 | serum screen | serum | n=704 TB  n=173 sPTB | 1^st^ TM | <37 | **0.3 (0.13-0.91)** | 95^th^ centile |
|  |  |  |  |  | 2^nd^ TM |  | **0.3 (0.09-0.78)** |  |
|  | Spencer et al 2008 | serum screen | serum | n=50,530 TB  n=3132 sPTB | 11-13 | <37 | **1.18** | 5^th^ centile |
|  |  |  |  | n=50,530 TB  n=1060 sPTB |  | <34 | **1.08** |  |
| AFP | Alleman et al 2014 | serum screen | serum | n=2499 TB  n=153 sPTB | 15-24 | <37 | **6.27 (2.93-13.4)** | 2-2.5 MoM |
|  | Beta et al 2011 | serum screen | serum | n=99 TB  n=33 sPTB | 11-13 | <34 | **s but no OR** |  |
|  | Goldenberg et al 2001 | serum screen | serum | n=50 TB  n=50 sPTB | 24 | <32 | **8.3** | 90^th^ centile |
|  |  |  |  | n=127 TB  n=127 sPTB |  | <35 | **3.5** |  |
|  | Jelliffe-Pawlowski et al 2010 | serum screen | serum | n=94,820 TB  n=1116 sPTB | 15-20 | <32 | **5.4 (4.4-6.7)** | >2 MoM |
|  |  |  |  | n=94,820 TB  n=6933 sPTB |  | 33-36 | **2.6 (2.4-2.9)** |  |
|  |  |  |  | n=94,820 TB  n=8049 sPTB |  | <37 | **2.8 (2.6-3.1)** |  |
|  | Jelliffe-Pawlowski et al 2013 | serum screen | serum | n=1725 TB  n=345 sPTB | 15-20 | <30 | **2.8 (1.7-4.6)** | 95^th^ centile |
|  | Jelliffe-Pawlowski et al 2015 | serum screen | serum | n= 784,064 TB  n= 30,579 sPTB | 15-20 | <31 | **3.5 (2.7-4.6)** | 95^th^ centile |
|  |  |  |  |  |  | 32-36 | **2.2 (1.9-2.5)** |  |
|  | McLean et al 1999 | radioimmunoassay | plasma | n=800 TB  n=37 sPTB | 17-30 | <37 | **1.9** | >2.1 MoM |
|  | Smith et al 2006 | serum screen | serum | n=79,116 TB  n=3017 sPTB | 15-24 | <37 | **2.44 (2.12-2.81)** | 95^th^ centile |
|  | Tripathi et al 2014 | ELISA | serum | n=137 TB  n=137 sPTB | 24-27 | <35 | 0.7016 (0.316-1.5576) | 90^th^ centile |
| estriol | Alleman et al 2013 | serum screen | serum | n=2499 TB  n=153 sPTB | 15-24 | <37 | ns |  |
|  | Jelliffe-Pawlowski et al 2010 | serum screen | serum | n=94,820 TB  n=1116 sPTB | 15-20 | <32 | **3.6 (2.0-6.7)** | >2 MoM |
|  |  |  |  | n=6933 sPTB |  | 33-36 | **3.7 (3.0-4.4)** |  |
|  |  |  |  | n=8049 sPTB |  | <37 | **3.5 (2.9-4.2)** |  |
|  | Jelliffe-Pawlowski et al 2013 | serum screen | serum | n=1725 TB  n=345 sPTB | 15-20 | <30 | 1.6 (0.9-2.8) | 95^th^ centile |
|  | Jelliffe-Pawlowski et al 2015 | serum screen | serum | n= 784,064 TB  n= 30,579 sPTB | 15-20 | <31 | 1.2 (0.8-1.8) | 95^th^ centile |
|  |  |  |  |  |  | 32-36 | 1.3 (1.1-1.5) |  |
|  | Olsen et al 2014 | immunoassay | serum | n=6478 TB  n=499 sPTB | 15-22 | <37 | **1.68 (1.41-2.02)** | >1 MoM |

ns denotes studies that reported no significant differences between the spontaneous preterm birth (sPTB) and term birth (TB) groups, and as such no odds ratios (OR) were calculated. S denotes that the study reported significant (p<0.05) differences between the sPTB and TB groups but did not subsequently report an OR. Bold font indicates those statistically significant OR (p<0.05). When multiple cut-offs were assessed, the odds ratio associated with the most extreme measured centile was recorded. TM: trimester, PPROM: preterm premature rupture of membranes.

**Table 2. Odds Ratios of sPTB Against Maternal Blood Levels of CRH**

| **Study** | **Method** | **Tissue** | **Sample Size** | **Collection**  **(weeks)** | **Outcome**  **(weeks)** | **OR (95% CI)** | **Cutoff** |
| --- | --- | --- | --- | --- | --- | --- | --- |
| Goldenberg et al 2001 | serum screen | serum | n=50 TB  n=50 sPTB | 24 | <32 | 2.7 | 90^th^ centile |
|  |  |  | n=127 TB  n=127 sPTB |  | <35 | 1.5 |  |
| Leung et al 1999 | radioimmunoassay | plasma | n=632 TB  n=11 sPTB | 15-20 | <34 | **9.4 (2.5-35.0)** | >1.9 MoM |
| McLean et al 1999 | radioimmunoassay | plasma | n=800 TB  n=37 sPTB | 17-30 | <37 | 4.6 | >2.6 MoM |
| Ruiz et al 2002 | radioimmunoassay | plasma | n=70 TB  n=6 sPTB | 15-19 | <37 | **s but no OR** |  |
|  |  |  |  | 20-22 |  | **s but no OR** |  |
|  |  |  |  | 23-26 |  | **s but no OR** |  |
|  |  |  |  | 27-30 |  | **s but no OR** |  |
| Sibai et al 2005 | ELISA | plasma | n=109 TB  n=61 sPTB | 16-20 | <37 | **s but no OR** |  |
|  |  |  | n=109 TB  n=33 sPTB |  | <35 | ns |  |

ns denotes studies that reported no significant differences between the spontaneous preterm birth (sPTB) and term birth (TB) groups, and as such no odds ratios (OR) were calculated. S denotes that the study reported significant (p<0.05) differences between the sPTB and TB groups but did not subsequently report an OR. Bold font indicates those statistically significant OR (p<0.05). When multiple cut-offs were assessed, the odds ratio associated with the most extreme measured centile was recorded.

**Table 3. Odds Ratios of sPTB Against Maternal Blood Levels of Inflammatory Biomarkers**

| **Biomarker** | **Study** | **Method** | **Tissue** | **Sample Size** | **Collection (weeks)** | **Outcome (weeks)** | **OR (95% CI)** | **Cut-off** |
| --- | --- | --- | --- | --- | --- | --- | --- | --- |
| CRP | Aung et al 2019 | ELISA | plasma | n=115 TB  n=31 sPTB | 23-29 | <37 | ̶-0.25 (-0.69-0.19) |  |
|  | Bakalis et al 2012 | immunoassay | serum | n=90 TB  n=30 sPTB | 11-13 | <34 | ns |  |
|  | Bullen et al 2013 | ELISA | plasma | n=992 TB  n=318 sPTB | 16-27 | <37 | ns |  |
|  | Catov et al 2014 | ELISA | serum | n=222 TB  n=115 sPTB | 4-20 | <37 | **2.02 (1.13-3.48)** | 66^th^ centile |
|  |  |  |  | n=82 sPTB |  | 34-36 | 1.03 (0.90-3.27) |  |
|  |  |  |  | n=33 sPTB |  | <34 | **3.73 (1.33-10.44)** |  |
|  | Ferguson et al 2014 | ELISA | plasma | n=350 TB  n=56 sPTB | 4.7-16.1 | <37 | 1.11 (0.82-1.50) |  |
|  |  |  |  |  | 14.9-21.9 |  | 1.31 (0.93-1.83) |  |
|  |  |  |  |  | 22.9-29.3 |  | 0.86 (0.62-1.19) |  |
|  |  |  |  |  | 33.1-38.3 |  | 1.07 (0.64-1.77) |  |
|  | Goldenberg et al 2001 | serum screen | serum | n=50 TB  n=50 sPTB | 24 | <32 | 1.7 | 90^th^ centile |
|  |  |  |  | n=127 TB  n=127 sPTB |  | <35 | 1.3 |  |
|  | Hvilsom et al 2002 | ELISA | serum | n=400 TB  n=84 sPTB | 14-19 | <37 | 1.6 (0.7-3.9) | 95^th^ centile |
|  | Khambalia et al 2015 | ELISA | serum | n=2079 TB  n=63 sPTB | 12 | <37 | **s but no OR** |  |
|  | Pitiphat et al 2005 | immunoasay | plasma | n=117 TB  n=117 sPTB | 5-19 | <37 | 7.24 (0.85-61.53) | >12 mg/L |
|  | Shin et al 2016 | immunoassay | serum | n=48 TB  n=24 sPTB | 11-18 | <37 | ns |  |
|  | Zhu et al 2018 | ELISA | plasma | n=556  n=40 sPTB | 11-14 | <34 | **s but no OR** |  |
| IL-6 | Aung et al 2019 | ELISA | plasma | n=115 TB  n=31 sPTB | 23-29 | <37 | 0.32 (-0.12-0.75) |  |
|  | Curry et al 2007 | flow cytometry | plasma | n=1125 TB  n=61 sPTB | 25 | 24-29 | 1.37 (0.63-2.94) | 90^th^ centile |
|  |  |  |  | n=278 sPTB |  | 30-33 | 1.26 (0.84-1.90) |  |
|  |  |  |  | n=334 sPTB |  | 34-36 | 1.41 (0.97-2.04) |  |
|  | Curry et al 2009 | flow cytometry | plasma | n=1372 TB  n=107 sPTB | 8 | 24-29 | 1.32 (0.72-2.44) | 90^th^ centile |
|  |  |  |  | n=353 sPTB |  | 30-33 | 1.34 (0.92-1.96) |  |
|  |  |  |  | n=422 sPTB |  | 34-36 | 1.38 (0.97-1.97) |  |
|  | Ferguson et al 2014 | ELISA | plasma | n=350 TB  n=56 sPTB | 4.7-16.1 | <37 | **1.22 (1.02-1.45)** |  |
|  |  |  |  |  | 14.9-21.9 |  | **1.29 (1.06-1.56)** |  |
|  |  |  |  |  | 22.9-29.3 |  | **1.25 (1.01-1.55)** |  |
|  |  |  |  |  | 33.1-38.3 |  | 1.21 (0.93-1.56) |  |
|  | Goldenberg et al 2001 | serum screen | serum | n=50 TB  n=50 sPTB | 24 | <32 | **3.9** | 90^th^ centile |
|  |  |  |  | n=127 TB  n=127 sPTB |  | <35 | 1.1 |  |
|  | Jelliffe-Pawlowski et al 2018 | immunoassay | serum | n=200 TB  n=200 sPTB | 15-20 | <37 | 0.83 (0.58-1.17) |  |
|  | Paternoster et al 2002 | immunoassay | serum | n=196 TB  n=29 sPTB | 24 | <37 | ns |  |
|  | Shin et al 2016 | immunoassay | serum | n=48 TB  n=24 sPTB | 11-18 | <37 | ns |  |
|  | Tripathi et al 2014 | ELISA | serum | n=137 TB  n=137 sPTB | 24-27 | <35 | 0.662 (0.3053-1.4354) | 90^th^ centile |
|  | Vogel et al 2007 | bead-based assay | serum | n=42 TB  n=15 sPTB | 12-15 | <35 | 3.5 (1.6-7.5) | >30pg/mL |
|  |  |  |  | n=120 sPTB |  | <37 | 2.3 (1.2-4.7) |  |
|  | Zhu et al 2018 | ELISA | plasma | n=556 TB  n=40 sPTB | 11-14 | <34 | **s but no OR** |  |
| TNFa | Aung et al 2019 | ELISA | plasma | n=115 TB  n=31 sPTB | 23-29 | <37 | ̶̶-0.06 (-0.46-0.34) |  |
|  | Curry et al 2007 | flow cytometry | plasma | n=1125 TB  n=61 sPTB | 25 | 24-29 | 0.99 (0.42-2.34) | 90^th^ centile |
|  |  |  |  | n=278 sPTB |  | 30-33 | 1.35 (0.90-2.01) |  |
|  |  |  |  | n=334 sPTB |  | 34-36 | 0.93 (0.61-1.41) |  |
|  | Curry et al 2009 | flow cytometry | plasma | n=1372 TB  n=107 sPTB | 8 | 24-29 | 0.79 (0.38-1.63) | 90^th^ centile |
|  |  |  |  | n=353 sPTB |  | 30-33 | 1.22 (0.83-1.79) |  |
|  |  |  |  | n=422 sPTB |  | 34-36 | **1.48 (1.05-2.09)** |  |
|  | Ferguson et al 2014 | ELISA | plasma | n=350 TB  n=56 sPTB | 4.7-16.1 | <37 | 1.08 (0.77-1.53) |  |
|  |  |  |  |  | 14.9-21.9 |  | 1.13 (0.78-1.64) |  |
|  |  |  |  |  | 22.9-29.3 |  | 1.17 (0.79-1.73) |  |
|  |  |  |  |  | 33.1-38.3 |  | 1.23 (0.78-1.95) |  |
|  | Huang et al 2019 | immunoassay | serum | n=258 TB  n=129 sPTB | 13-28 | <36 | **2.84 (1.28-6.29)** | >69.73pg/mL |
|  | Jelliffe-Pawlowski et al 2018 | immunoassay | serum | n=200 TB  n=200 sPTB | 15-20 | <37 | 0.97 (0.79-1.21) |  |
|  | Paternoster et al 2002 | immunoassay | serum | n=196 TB  n=29 sPTB | 24 | <37 | ns |  |
|  | Shin et al 2016 | immunoassay | serum | n=48 TB  n=24 sPTB | 11-18 | <37 | ns |  |
|  | Vogel et al 2007 | bead-based assay | serum | n=42 TB  n=15 sPTB | 12-15 | <35 | **4.9 (2.4-10.0)** | >165pg/mL |
|  |  |  |  | n=120 sPTB |  | <37 | **3.1 (1.7-5.4)** |  |
| IL-10 | Aung et al 2019 | ELISA | plasma | n=115 TB  n=31 sPTB | 23-29 | <37 | 0.20 (-0.20-0.60) |  |
|  | Ferguson et al 2014 | ELISA | plasma | n=350 TB  n=56 sPTB | 4.7-16.1 | <37 | 1.17 (0.94-1.46) |  |
|  |  |  |  |  | 14.9-21.9 |  | 1.26 (0.98-1.60) |  |
|  |  |  |  |  | 22.9-29.3 |  | **1.36 (1.05-1.78)** |  |
|  |  |  |  |  | 33.1-38.3 |  | 1.08 (0.80-1.46) |  |
|  | Goldenberg et al 2001 | serum screen | serum | n=50 TB  n=50 sPTB | 24 | <32 | 0.5 | 90^th^ centile |
|  |  |  |  | n=127 TB  n=127 sPTB |  | <35 | 0.4 |  |
|  | Huang et al 2019 | immunoassay | serum | n=258 TB  n=129 sPTB | 13-28 | <36 | 2.56 (1.03-6.37) | >20.16 pg/mL |
|  | Jelliffe-Pawlowski et al 2018 | immunoassay | serum | n=200 TB  n=200 sPTB | 15-20 | <37 | 0.99 (0.79-1.25) |  |
|  | Shin et al 2016 | immunoassay | serum | n=48 TB  n=24 sPTB | 11-18 | <37 | ns |  |
|  | Vogel et al 2007 | bead-based assay | serum | n=42 TB  n=15 sPTB | 12-15 | <35 | 3.1 (0.96-9.7) | >320 pg/mL |
|  |  |  |  | n=120 sPTB |  | <37 | 1.4 (0.6-2.8) |  |
| IL-2 | Curry et al 2007 | flow cytometry | plasma | n=1125 TB  n=61 sPTB | 25 | 24-29 | 1.17 (0.52-2.64) | 90^th^ centile |
|  |  |  |  | n=278 sPTB |  | 30-33 | 0.97 (0.63-1.51) |  |
|  |  |  |  | n=334 sPTB |  | 34-36 | 1.20 (0.81-1.76) |  |
|  | Curry et al 2009 | flow cytometry | plasma | n=1372 TB  n=107 sPTB | 8 | 24-29 | 0.88 (0.43-1.83) | 90^th^ centile |
|  |  |  |  | n=353 sPTB |  | 30-33 | 1.14 (0.77-1.70) |  |
|  |  |  |  | n=422 sPTB |  | 34-36 | 1.08 (0.74-1.58) |  |
|  | Jelliffe-Pawlowski et al 2018 | immunoassay | serum | n=200 TB  n=200 sPTB | 15-20 | <37 | 1.00 (0.78-1.29) |  |
|  | Shin et al 2016 | immunoassay | serum | n=48 TB  n=24 sPTB | 11-18 | <37 | ns |  |
|  | Vogel et al 2007 | bead-based assay | serum | n=42 TB  n=15 sPTB | 12-15 | <35 | **3.3 (1.5-7.0)** | >120pg/mL |
|  |  |  |  | n=120 sPTB |  | <37 | **2.0 (1.0-4.0)** |  |
| G-CSF | Goldenberg et al 2001 | unknown | serum | n=50 TB  n=50 sPTB | 24 | <32 | **5.5** | 75^th^ centile |
|  |  |  |  | n=127 TB  n=127 sPTB |  | <35 | 1 |  |
|  | Goldenberg et al 2000 | ELISA | plasma | n=194 TB  n=194 sPTB | 24 | <28 | **4.5 (1.3-16.0)** | 75^th^ centile |
|  |  |  |  |  |  | 29-31 | **8.1(1.5-42.8)** |  |
|  |  |  |  |  |  | 32-34 | 1.5 (0.6-3.9) |  |
|  |  |  |  |  |  | 35-36 | 0.6 (0.3-1.1) |  |
|  |  |  |  |  | 28 | 28-31 | **10.5 (1.1-499)** |  |
|  |  |  |  |  |  | 32-34 | 0.7 (0.3-1.6) |  |
|  |  |  |  |  |  | 35-36 | 0.8 (0.3-1.7) |  |
|  | Jelliffe Pawlowski et al 2018 | immunoassay | serum | n=200 TB  n=200 sPTB | 15-20 | <37 | 1.48 (0.81 -2.70) |  |
|  | Tripathi et al 2014 | ELISA | serum | n=137 TB  n=137 sPTB | 24-27 | <35 | 0.9205 (0.4505-1.8807) | 90^th^ centile |
|  | Whitcomb et al 2009 | immunoassay | serum | n=394 TB  n=31 sPTB | 6-18 | <37 | **1.52 (1.07-2.16)** |  |
| IFNy | Curry et al 2007 | flow cytometry | plasma | n=1125 TB  n=61 sPTB | 25 | 24-29 | 0.81 (0.32-2.06) | 90^th^ centile |
|  |  |  |  | n=278 sPTB |  | 30-33 | **1.56 (1.07-2.30)** |  |
|  |  |  |  | n=334 sPTB |  | 34-36 | 1.27 (0.87-1.86) |  |
|  | Curry et al 2009 | flow cytometry | plasma | n=1372 TB  n=107 sPTB | 8 | 24-29 | 0.59 (0.27-1.33) | 90^th^ centile |
|  |  |  |  | n=353 sPTB |  | 30-33 | 0.74 (0.48-1.15) |  |
|  |  |  |  | n=422 sPTB |  | 34-36 | 0.74 (0.49-1.11) |  |
|  | Jelliffe-Pawlowski et al 2018 | immunoassay | serum | n=200 TB  n=200 sPTB | 15-20 | <37 | 1.01 (0.90-1.13) |  |
|  | Shin et al 2016 | immunoassay | serum | n=48 TB  n=24 sPTB | 11-18 | <37 | ns |  |
| IL-1B | Aung et al 2019 | ELISA | plasma | n=115 TB  n=31 sPTB | 23-29 | <37 | 0.15 (-0.27-0.58) |  |
|  | Ferguson et al 2014 | ELISA | plasma | n=350 TB  n=56 sPTB | 4.7-16.1 | <37 | 1.03 (0.86-1.23) |  |
|  |  |  |  |  | 14.9-21.9 |  | 0.97 (0.80-1.17) |  |
|  |  |  |  |  | 22.9-29.3 |  | 1.05 (0.87-1.26) |  |
|  |  |  |  |  | 33.1-38.3 |  | 1.04 (0.83-1.29) |  |
|  | Jelliffe-Pawlowski et al 2018 | immunoassay | serum | n=200 TB  n=200 sPTB | 15-20 | <37 | 1.00 (0.88-1.13) |  |
|  | Vogel et al 2007 | bead-based assay | serum | n=42 TB  n=15 sPTB | 12-15 | <35 | **3.5 (1.6-7.7)** |  |
|  |  |  |  | n=42 TB  n=120 sPTB |  | <37 | **2.5 (1.3-4.7)** |  |
| ferritin | Abdel Malek et al 2018 | ELISA | serum | n=196 TB  n=40 sPTB | 30 | <37 | s but no OR |  |
|  | Beta et al 2012 | immunoassay | serum | n=90 TB  n=30 sPTB | 11-13 | <34 | ns |  |
|  | Goldenberg et al 2001 |  | serum | n=50 TB  n=50 sPTB | 24 | <32 | 8 | 90^th^ centile |
|  |  |  |  | n=127 TB  n=127 sPTB |  | <35 | 1.4 |  |
|  | Khambalia et al 2015 | ELISA | serum | n=2079 TB  n=63 sPTB | 12 | <34 | **2.54 (1.36-4.67)** | 90^th^ centile |
|  |  |  |  | n=112 sPTB |  | 34-36 | **1.75 (1.02, 2.99)** |  |
|  | Movahedi et al 2012 | immunoassay | serum | n=153 TB  n=69 sPTB |  | <37 | s but no OR |  |
|  | Paternoster et al 2002 | immunoassay | serum | n=196 TB  n=29 sPTB | 24 | <37 | 2.12 (0.6-7.5) | 90^th^ centile |
|  |  |  |  | n=19 sPTB |  | <32 | 1.2 (0.2-6.1) |  |
|  |  |  |  | n=25 sPTB |  | <35 | **4.85 (1.6-14.7)** |  |
|  | Tripathi et al 2014 | ELISA | serum | n=137 TB  n=137 sPTB | 24-27 | <35 | 0.464 (0.2089-1.0333) | 90^th^ centile |

ns denotes studies that reported no significant differences between the spontaneous preterm birth (sPTB) and term birth (TB) groups, and as such no odds ratios (OR) were calculated. s denotes that the study reported significant (p<0.05) differences between the sPTB and TB groups but did not subsequently report an OR. Bold font indicates those statistically significant OR (p<0.05). When multiple cut-offs were assessed, the odds ratio associated with the most extreme measured centile was recorded.
